# The resilience of weed seedbank regulation by carabid beetles, at continental scales, to alternative prey

**DOI:** 10.1038/s41598-020-76305-w

**Published:** 2020-11-09

**Authors:** Benjamin Carbonne, Sandrine Petit, Veronika Neidel, Hana Foffova, Eirini Daouti, Britta Frei, Jiří Skuhrovec, Milan Řezáč, Pavel Saska, Corinna Wallinger, Michael Traugott, David A. Bohan

**Affiliations:** 1 Agroécologie, AgroSup Dijon, INRAE, Université de Bourgogne Franche-Comté, 17 rue Sully, BP 86510, 21065 Dijon Cedex, France; 2 Mountain Agriculture Research Unit, Institute of Ecology, University of Innsbruck, Innsbruck, Austria; 3 Functional Diversity in Agro-Ecosystems, Crop Research Institute, Drnovská 507, Ruzyně, 161 06 Praha 6, Czech Republic; 4 Department of Ecology, Faculty of Environmental Sciences, Czech University of Life Sciences Prague, Kamýcká 129, Suchdol, 165 00 Praha, Czech Republic; 5 Department of Ecology, Swedish University of Agricultural Sciences, Box 7044, 75007 Uppsala, Sweden

**Keywords:** Agroecology, Ecosystem services, Ecology

## Abstract

Carabids are generalist predators that contribute to the agricultural ecosystem service of seedbank regulation via weed seed predation. To facilitate adoption of this ecosystem services by farmers, knowledge of weed seed predation and the resilience of seedbank regulation with co-varying availability of alternative prey is crucial. Using assessments of the seedbank and predation on seed cards in 57 cereal fields across Europe, we demonstrate a regulatory effect on the soil seedbank, at a continental scale, by groups formed of omnivore, seed-eating (granivore + omnivore) and all species of carabids just prior to the crop-harvest. Regulation was associated with a positive relationship between the activity-density of carabids and seed predation, as measured on seed cards. We found that per capita seed consumption on the cards co-varied negatively with the biomass of alternative prey, i.e. Aphididae, Collembola and total alternative prey biomass. Our results underline the importance of weed seedbank regulation by carabids, across geographically significant scales, and indicate that the effectiveness of this biocontrol may depend on the availability of alternative prey that disrupt the weed seed predation.

## Introduction

The regulation of weeds by natural enemies is an important ecosystem service in arable farmland^1^ that might be used to reduce herbicide applications^2^. Carabids are abundant predators in arable fields that have been shown to consume a wide range of species of weed seeds under laboratory conditions^3–5^ and consume large numbers of seeds^6–9^ in field studies. Under field conditions this weed seed consumption by carabids can regulate the growth of weed populations^10,11^. Foraging carabids intercept and consume weed seeds that have fallen onto the soil surface, during a window of opportunity before they then enter the soil and join the seedbank^12,13^. This interception and predation reduces the number of seeds that can then enter the seedbank, thus modifying the size of the seedbank. Where carabid abundance and seed consumption is high enough, carabid predation may regulate the seedbank, potentially reducing future weed emergence. In-field predation of weed seeds by carabids^11,14,15^ is typically estimated using seed predation cards^16^, in which a standard number of seeds of a particular weed species are presented at the soil surface to estimate seed removal as a proxy metric for seed predation. Evidence from the field of weed regulation is relatively scarce and limited to studies that have assessed predator effects on weed seedling emergence^17,18^ or the weed seedbank^19^. Bohan et al.^19^ found a negative relationship between yearly weed seedbank turnover and carabid counts, which would indicate a regulatory effect of carabids on weeds at the UK national scale.

One obstacle to the adoption of this specific ecosystem service in farming has been the great variability of weed seed predation estimates between studies^20–23^. This variation could be caused by the consumption of alternative prey instead of seeds. Carabid communities are often classified into trophic guilds, such as granivores, omnivores and carnivores that forage at the soil surface and occasionally climb into the vegetation^24^. The majority of species in these guilds feed on both plant and animal food^9^, including seeds, alternative animal prey such as slugs, aphids, earthworms and springtails^25–27^, and intraguild prey such as spiders and other carabids. Given their broad dietary range, the effectiveness of carabids in regulating weeds will likely co-vary with the local availability and biomass of alternative prey^28–31^. In the ‘alternative prey hypothesis’, alternative prey and weed seeds may interact indirectly via their shared natural enemies either positively (e.g. apparent mutualism or commensalism)^31^ or negatively (e.g. apparent competition or amensalism)^31^. Positive indirect interactions between weed seeds and alternative prey would lead to a decrease in the effectiveness of weed seed predation by carabids, thus potentially disrupting weed regulation. The indirect trophic mechanisms by which the effectiveness of weed predation might decline, caused by alternative prey, would include carabid satiety, switching behaviour, prey preference or a dilution of encounter rates between the seeds and the carabids. Following the outcomes of laboratory studies that demonstrate alternative prey disruption of the biological control of aphids, slugs and dipteran eggs^32–36^, we expect that carabid predation of weed seeds will also vary with the availability of alternative prey, affecting the in-field regulation of weeds. In turn, we expect the effect of alternative prey on seed predation by carabids to vary over the crop-growing period, as a result of variation in the diet preferences of carabids and changes in the relative availability of alternative prey to seeds^37,38^.

The outcomes of studies on in-field weed seed predation intensity of carabids are inconsistent^20,21^ and appear context-dependent^22,23^. We lack both the large-scale replicated data for the contribution of carabid seed predation to weed regulation and the understanding of the trophic mechanisms underlying context dependency and resilience of weed seed predation and regulation^19,20^. Specifically, there is a knowledge gap for the role of the availability of alternative prey and how it affects weed seed predation by carabids across the crop-growing period. Here, we test for the context-dependency of seed predation to presence of alternative prey and for the generality of regulation across a highly replicated, large-scale study of 57 cereal fields in four EU countries (Austria, the Czech Republic, France and Sweden). We tested four expectations, such that: (i) there is a negative relationship between the change in the size of the weed seedbank over a year and the activity-density (AD) of carabids, indicative of weed seedbank regulation. The AD of carabids corresponds to the number of individuals captured using pitfall traps, which depends both on their activity on the ground surface and their density. (ii) The observed weed seedbank regulation relationship co-varies with the availability of alternative prey biomass; (iii) the relationship between the AD of carabids and weed seed predation, as evaluated using seed predation cards, will be significant and positive; and, (iv) the local availability of alternative prey biomass will be a significant co-variate of the per capita seed consumption by carabids, using seed cards. Carabid AD, seed card predation and alternative prey biomass were measured during the growing season and just prior to harvest, in each country, allowing us to test the hypotheses at two key moments during the cropping cycle. Carabid AD was described and tested for the groups of all carabid species, seed-eating (granivore + omnivore), granivore and omnivore that can respond differently to alternative prey. In addition, the proportion of arable crops in the landscape and the pesticide management intensity were both used as co-variables in our analyses as they could counfound explainations of weed seed predation and seedbank change.

## Results

### Carabid counts in pitfall traps

A total of 70,545 carabids (71,104 after standardization for missing traps) belonging to 117 species was caught during the field survey, with a mean AD of 593 (SD = 487) carabids per field and session, and a mean species richness of 15.8 (SD = 4.69) per field and session (detail per country in Supp. Mat. Table S1 and Fig. S1 after standardization for missing traps). In Austria, there were on average 985 (647) carabids per field and session. This was a greater number of carabids per field than in the other countries, with 432 (313) being trapped in the Czech Republic, 383 (293) in France and 570 (374) in Sweden. The dominant species differed between countries, but the 10 most abundant species were similar (Supp. Mat. Table S2 and Fig. S2). Omnivores were the dominant carabid guild (42,401 individuals in total), with a species dominance of *Pterostichus melanarius* (Illiger, 1798) (17,183 individuals in total) in the Czech Republic and Sweden, and *Poecilus cupreus* (Linnaeus, 1758) (17,105 individuals in total) in France and Austria. The omnivores were followed in importance by carnivores (23,671 carabids), which were mainly represented by *Anchomenus dorsalis* (Pontopppidan, 1763) (10,501 individuals). The granivore guild was the least abundant (5 032 individuals in total), and was dominated by *Harpalus rufipes* (De Geer, 1774) (2140 individuals), *Harpalus affinis* (Schrank, 1781) (998 individuals) and *Amara similata* (Gyllenhal, 1810) (653 individuals). Overall, slightly more granivore carabids were caught in the second sampling session. In particular, there was an increase in the AD of granivore carabids per field in France and Austria between the session 1 and 2, but a decrease in Sweden (Supp. Mat. Fig. S1).

### Seedbank and seed cards

A total of 14,381 seeds was sampled in the initial seedbank and 14,315 seeds in the follow-up seedbanks, made up of 174 plant taxa. The amount of seeds in both initial and follow-up seedbank was unbalanced between countries, with a higher abundance of seeds and richness of plant species in Austria and Sweden than in France, with an intermediate level in the Czech Republic (Supp. Mat. Table S3 and Fig. S3). The mean predation rate, per field, of *Poa annua* L. seeds ranged from 0.87% to 90.1% and was 16.1% on average over all countries (Austria: 18.7%, the Czech Republic: 23.8%, France: 9.5% and Sweden: 12.5%), which corresponded to 128 seeds eaten per field on average on seed cards (Supp. Mat. Table S4 and Fig. S4). The seed predation rate per field was higher during the session 1 (20.5%) than the session 2 (11.7%) on average. The average per capita seed consumption per field and session varied by carabid group, with on average 0.88 (SD = 2.23) seeds consumed per omnivore and 7.88 (SD = 10.2) per granivore (Supp. Mat. Table S5). The average per capita seed consumption was lower in session 2 than in session 1, irrespective of the carabid group considered.

### Alternative prey

The alternative prey samples were dominated by Aphididae (394 individuals per field and session, biomass = 1770 mg) and Collembola (2400 individuals per field and session, 198 mg). We also captured 4170 Arachnida (mostly Linyphiidae, Araneidae, Theridiidae and Lycosidae), equating to 39.5 individuals (40.9 mg) per field and session. The total biomass of alternative prey (sum of Aphididae, Collembola and Arachnida) was an average of 2010 mg per field, with higher biomass being sampled in Austria and Sweden than in the Czech Republic and in France (Supp. Mat. Table S7 and S8). There was a reduction in the total biomass of alternative prey sampled between session 1 and 2 in France and Sweden (on average − 294 mg and − 3240 mg per field, respectively), but an increase in the Czech Republic (on average + 656 mg of total biomass per field). These three groups of prey were used as indicators of the alternative prey availability for the analysis because they represented 83.4% of all individual arthropods captured (70.6% Collembola, 11.6% Aphididae and 1.2% Arachnida) and are also known to be consumed by carabids^25–27^. Other abundant arthropod groups, such as Thysanoptera (4.7%), Hymenoptera (other than ants, 2.7%), Nematocera (2.1%), Acari (1.5%), Auchenorrhyncha (1.3%) and Brachycera (1.2%), were not analysed because of the lack of evidence that they are frequently preyed upon by carabids^26,27^. The list of all identified alternative prey groups is presented in Suppl. Mat. Table S9.

### Models of expectation

#### (i) The relationship between the weed seedbank change and the AD of carabids

**Figure 1:**
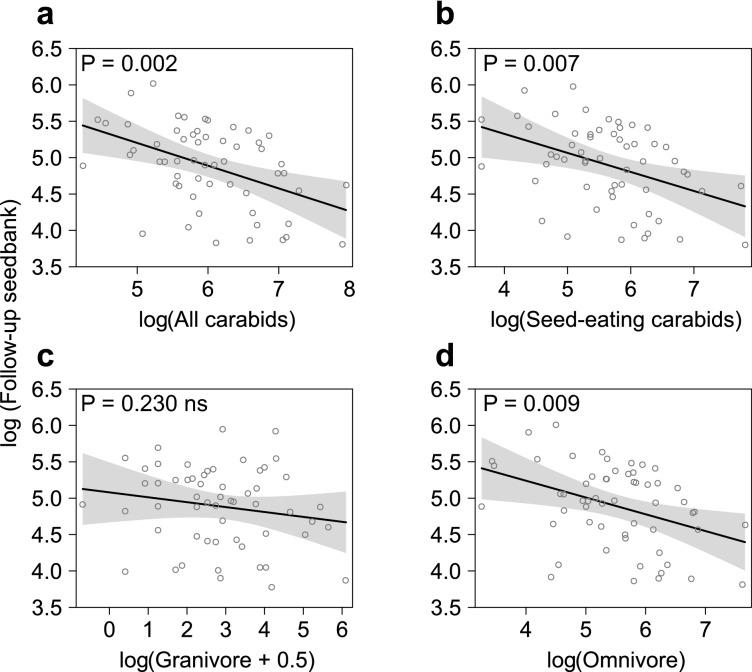
Multiple linear regression model fits to test the weed seedbank regulation by carabids. The log(Follow-up seedbank) is plotted against the log-transformed AD of: **(a) **all occurring carabid species; **(b)** seed-eating; **(c)** granivore; and, **(d)** omnivore carabids in session 2. The line represents the fixed-effect prediction and associated 95% confidence intervals (shaded), with the open circles being the partial residuals. Negative slopes indicate a regulatory effect of carabid AD on the seedbank.

An increase in AD of all carabids, totalled (linear model (LM): F_1,57_ = 10.89, P = 0.002), seed-eating carabids (LM: F_1,57_ = 7.82, P = 0.007) and omnivores (LM: F_1,57_ = 7.31, P = 0.009) was associated with a decrease in the follow-up seedbank during session 2 (Fig. 1, Table 1 and Supp. Mat. Table S10). No significant relationship was observed for any carabid group in session 1 (Supp. Mat. Table S10) or for the granivores.

**Table 1:** Results of linear models (LMs) relating the follow-up seedbank to the initial seedbank, the proportion of crop, the pesticide intensity and to the seed-eating carabid AD alone or in interaction with alternative prey biomass.

Analyses	Session	AIC	R^2^	Explanatory variable	Est (std. error)	F	df	P-value
Effect of SE	1 (n = 57)	103.3	0.68	log(Initial seedbank)	0.72 (0.08)	70.95	1	< 0.001***
				log(SE)	0 (0.10)	0.00	1	0.999
				PesticideIntensity	− 0.03 (0.05)	0.44	1	0.510
				pCrop	0.53 (0.42)	1.58	1	0.214
	2 (n = 57)	93.3	0.72	log(Initial seedbank)	0.76 (0.08)	97.06	1	< 0.001***
				log(SE)	− 0.26 (0.09)	7.82	1	0.007**
				PesticideIntensity	− 0.06 (0.05)	1.69	1	0.200
				pCrop	0.33 (0.40)	0.69	1	0.412
Effect of SE carabids × aphids	1 (n = 57)	98.8	0.72	log(SE):log(Aphid)	0.01 (0.08)	0.01	1	0.922
				log(SE)	− 0.01 (0.49)	0.18	1	0.676
				log(Aphid)	− 0.21 (0.46)	8.05	1	0.007**
				log(Initial seedbank)	0.74 (0.08)	75.93	1	< 0.001***
				PesticideIntensity	− 0.08 (0.05)	2.01	1	0.162
				pCrop	0.68 (0.41)	2.77	1	0.102
	2 (n = 42)	73.4	0.75	log(SE):log(Aphid)	− 0.01 (0.18)	0.00	1	0.966
				log(SE)	− 0.13 (1.07)	2.00	1	0.166
				log(Aphid)	0.06 (0.96)	0.02	1	0.877
				log(Initial seedbank)	0.73 (0.09)	66.65	1	< 0.001***
				PesticideIntensity	− 0.10 (0.06)	2.92	1	0.097
				pCrop	− 0.23 (0.52)	0.20	1	0.660
Effect of SE carabids × collembola	1 (n = 57)	105.5	0.68	log(SE):log(Collembola + 0.5)	0.07 (0.05)	1.56	1	0.217
				log(SE)	− 0.36 (0.31)	0.00	1	0.955
				log(Collembola + 0.5)	− 0.44 (0.36)	0.01	1	0.917
				log(Initial seedbank)	0.69 (0.09)	59.56	1	< 0.001***
				PesticideIntensity	− 0.03 (0.05)	0.29	1	0.595
				pCrop	0.56 (0.48)	1.33	1	0.254
	2 (n = 42)	67.3	0.79	log(SE):log(Collembola + 0.5)	− 0.01 (0.07)	0.02	1	0.891
				log(SE)	− 0.10 (0.32)	1.41	1	0.242
				log(Collembola + 0.5)	0.15 (0.40)	5.52	1	0.025*
				log(Initial seedbank)	0.78 (0.08)	95.42	1	< 0.001***
				PesticideIntensity	− 0.16 (0.06)	8.03	1	0.008**
				pCrop	0.07 (0.49)	0.02	1	0.882
Effect of SE carabids × arachnida	1 (n = 57)	103	0.69	log(SE):log(Arachnida)	− 0.21 (0.14)	2.48	1	0.122
				log(SE)	0.60 (0.39)	0.00	1	0.999
				log(Arachnida)	1.3 (0.78)	1.37	1	0.247
				log(Initial seedbank)	0.68 (0.09)	61.53	1	< 0.001***
				PesticideIntensity	− 0.04 (0.06)	0.48	1	0.49
				pCrop	0.48 (0.42)	1.30	1	0.26
	2 (n = 42)	71.6	0.76	log(SE):log(Arachnida)	0.10 (0.12)	0.64	1	0.428
				log(SE)	− 0.55 (0.50)	1.80	1	0.189
				log(Arachnida)	− 0.40 (0.59)	0.91	1	0.347
				log(Initial seedbank)	0.73 (0.08)	75.46	1	< 0.001***
				PesticideIntensity	− 0.12 (0.05)	4.60	1	0.039*
				pCrop	0.02 (0.54)	0.00	1	0.978
Effect of SE carabids × total prey	1 (n = 57)	96.4	0.73	log(SE):log(Total animal)	0.18 0.13)	2.05	1	0.159
				log(SE)	− 1.23 (0.87)	0.00	1	0.965
				log(Total animal)	− 1.26 (0.75)	8.42	1	0.005**
				log(Initial seedbank)	0.72 (0.8)	82.58	1	< 0.001***
				PesticideIntensity	− 0.08 (0.05)	2.35	1	0.132
				pCrop	0.57 (0.39)	2.11	1	0.152
	2 (n = 42)	70.4	0.77	log(SE):log(Total animal)	0.13 (0.25)	0.29	1	0.594
				log(SE)	− 1.05 (1.61)	2.51	1	0.122
				log(Total animal)	− 0.49 (1.34)	2.34	1	0.135
				log(Initial seedbank)	0.69 (0.08)	67.17	1	< 0.001***
				PesticideIntensity	− 0.12 (0.06)	4.14	1	0.049*
				pCrop	− 0.03 (0.50)	0.00	1	0.946

Five different analysis were conducted to test the effect of seed-eating (SE) carabids alone on weed seedbank change and to assess the interaction effect of seed-eating (SE) carabids with alternative prey (Aphididae, Collembola, Arachnida and the total of alternative prey). We fitted models for sessions 1 and 2 separately. Results for the other carabid groups are presented in Supp. Mat. Table S10. For each model we report the corresponding session with the number of observation (n), the AIC, the R-squared (R^2^), and for each variable in the model we state the estimate with standard error (Est. (Std. Error)), the F-value (F), the degree of freedom (Df) and the P-value.

#### (ii) Weed seedbank regulation relationship will co-vary with the availability of alternative prey biomass

The biomass of alternative prey did not modulate the relationship between carabids and seedbank change, whether considered as Collembola, Aphididae, Arachnida or the total alternative prey biomass (Table 1 and Supp. Mat. Table S10). For the biomass of Arachnida in session 1, a marginally non-significant interaction with the AD of all carabid species when totalled was detected (LM: F_1,57_ = 3.01, P = 0.089), with the slope of seedbank change going from positive to negative as Arachnida biomass increased (Supp. Mat. Table S10 and Fig. S5).

#### (iii) The relationship between the activity-density of carabids and weed seed predation

**Figure 2:**
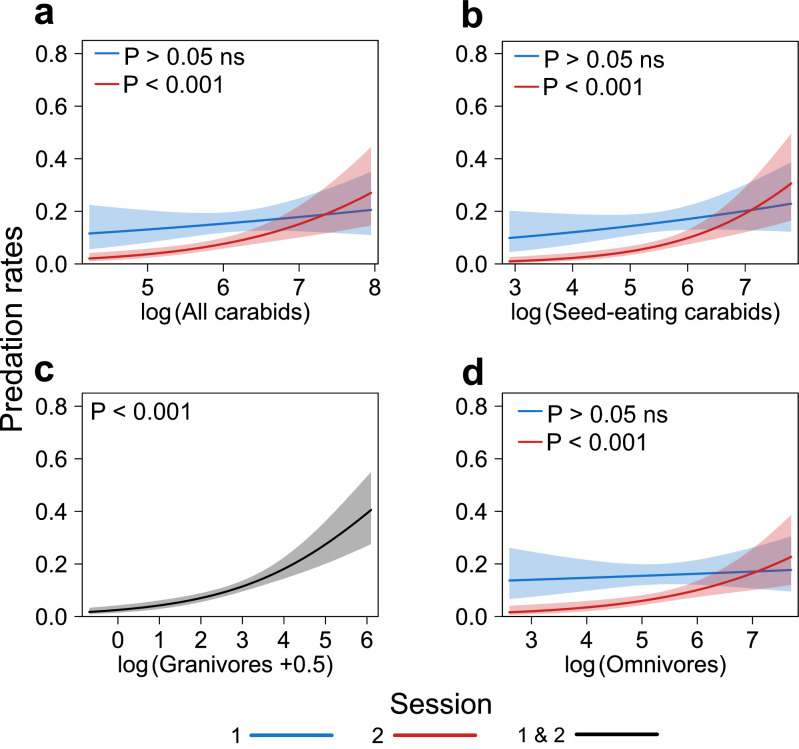
Fixed-effect predictions, with the associated 95% confidence intervals (blue, red or grey shaded), for the relationships between the predation rates of *Poa annua* on weed seed cards and the log-transformed activity-density (AD) of: **(a)** all-occurring carabid species; **(b)** seed-eating; **(c)** granivore; and, **(d)** omnivore carabids. Where the interaction between the AD of carabids and the session was significant, we present the relationships for the two sampling sessions (blue = session 1 (n = 58); red = session 2 (n = 58)), and we indicate whether the slope was different from 0 (P value for each session). For non-significant interactions, a single relationship is presented (black = session 1 & 2 (n = 116)).

The predation of *Poa annua* on seed cards was significantly affected by the interaction between the sampling session and the AD of all carabid species when totalled (generalised linear mixed model (GLMM): χ^2^_1,116_ = 5.63, P = 0.018), seed-eating carabids (GLMM: χ^2^_1,116_ = 7.81, P = 0.005) and omnivores (GLMM: χ^2^_1,116_ = 7.01, P = 0.008). For these three carabid groups, we detected a positive effect of AD on seed predation only during the session 2 (Table 2, Fig. 2, Supp. Mat. Table S11). The AD of granivore carabid species was significantly and positively associated with the seed predation rate (GLMM: χ^2^_1,116_ = 18.51, P < 0.001), for both sampling sessions (Supp. Mat. Table S11).

**Table 2:** Results of mixed linear models (GLMMs and LMMs) relating weed seed predation rates (based on seed cards count) to proportion of arable crops, pesticide intensity and activity-density (AD) of seed-eating (SE) carabids in interaction with session. Followed by models relating the number of seed eaten per seed-eating (SE) carabid to proportion of arable crops, pesticide intensity and alternative prey biomass in interaction with session.

Response variable (y)								
Model (n)	AIC	R^2^m	R^2^c	Explanatory variable (x)	Est. (std. error)	χ^2^	df	P-value
**Predation rates (seed cards)**								
GLMM (n = 116)	1333.6	0.10	0.34	log(SE):Session	S1: 0.20 (0.16); P > 0.05 ns	7.81	1	0.005**
					S2: 0.78 (0.18); P < 0.001***			
				log(SE)	0.20 (0.16)	1.73	1	0.189
				Session	S1: 0.16 (0.02)^a^; S2: 0.07 (0.01)^b^	12.30	1	< 0.001***
				PesticideIntensity	− 0.08 (0.07)	1.43	1	0.232
				pCrop	0.57 (0.20)	0.22	1	0.640
**Seed eaten per seed-eating carabid**								
LMM (n = 101)	300.1	0.23	0.40	log(Aphid):Session	− 0.23 (0.09)	3.79	1	0.052
				log(Aphid)	− 0.31 (010)	6.25	1	0.012*
				Session	S1: 0.49 (0.07)^a^; S2: 0.18 (0.03)^b^	27.40	1	< 0.001***
				PesticideIntensity	0.09 (0.76)	0.09	1	0.761
				pCrop	0.52 (0.62)	0.73	1	0.394
	290.8	0.28	0.63	log(Collembola + 0.5):Session	S1: 0.16 (0.07); P = 0.02	27.96	1	< 0.001***
					S2: − 0.18 (0.06); P = 0.01			
				log(Collembola + 0.5)	0.16 (0.07)	5.98	1	0.014*
				Session	S1: 0.47 (0.06); S2: 0.17 (0.03)	1.35	1	0.245
				PesticideIntensity	0.11 (0.07)	2.44	1	0.118
				pCrop	0.61 (0.73)	0.71	1	0.400
	309.2	0.17	0.32	log(Arachnida):Session	− 0.02 (0.11)	0.32	1	0.570
				log(Arachnida)	0.08 (0.21)	0.04	1	0.837
				Session	S1: 0.47 (0.07)^a^; S2: 0.19 (0.03)^b^	16.28	1	< 0.001***
				PesticideIntensity	0.08 (0.06)	1.45	1	0.229
				pCrop	0.37 (0.66)	0.31	1	0.575
	301.5	0.22	0.37	log(Total animal):Session	− 0.32 (0.11)	0.61	1	0.436
				log(Total animal)	− 0.36 (012)	7.77	1	0.005**
				Session	S1: 0.49 (0.07)^a^; S2: 0.18 (0.03)^b^	26.87	1	< 0.001***
				PesticideIntensity	0.04 (0.06)	0.43	1	0.511
				pCrop	0.40 (0.61)	0.44	1	0.507

For each model we reported the type of model with the number of observations (n), AIC, marginal and conditional R-squared (R^2^m and R^2^c), and for each variable in the model we report the estimate with standard error (est. (std. error)), the Wald Chi square-value (χ^2^), test degrees of freedom (df) and the P-value. Where the interaction between carabids AD or prey biomass with session was significant, we report the estimate with standard error (est. (std. error)) and indicate if the slope is different from 0 (P-value) for each sampling session. For qualitative factors, such as session (S1: session 1; S2: session 2) we report the estimated marginal means (std. error) and an associated letter indicating the significance of the difference between the two sessions.

#### (iv) The local availability of alternative prey biomass will be a significant co-variate of the per capita seed consumption by carabids

**Figure 3:**
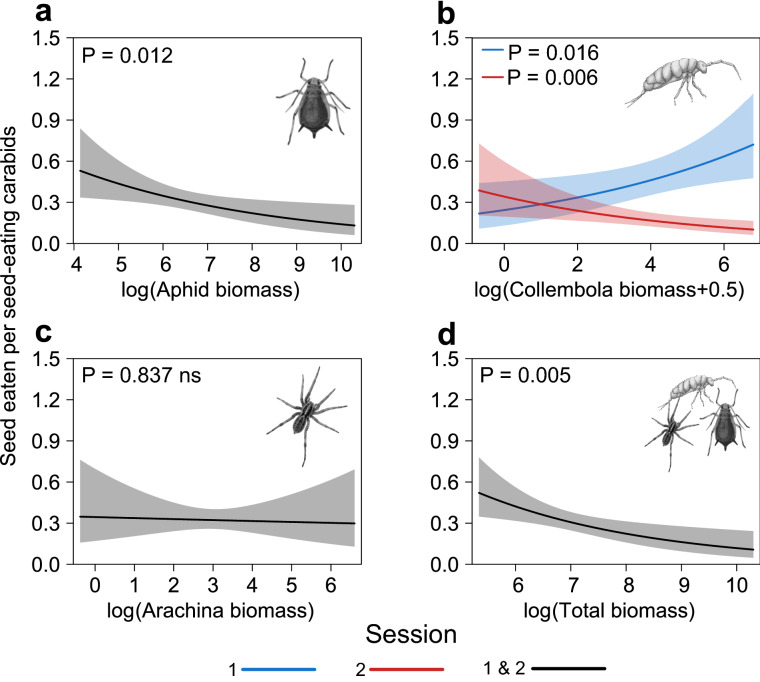
Fixed-effect predictions, with the associated 95% confidence intervals (blue, red or grey shaded), for the relationships between the log-transformed biomass of **(a)** Aphididae, **(b)** Collembola, **(c)** Arachnida and **(d)** total alternative prey and the number of seeds eaten, on seed cards, per seed-eating carabids. Where the interaction between the prey biomass and the session was significant, we present the relationships for the two sampling sessions (blue = session 1 (n = 58); red = session 2 (n = 43)), and we indicate whether the slope was different from 0 (P value for each session). For non-significant interactions, a single relationship is presented (black = session 1 & 2 (n = 101)).

An increase in the number of seeds eaten, on seed cards, per seed-eating carabid was significantly associated with a decrease in the Aphididae biomass (linear mixed model (LMM): χ^2^_1,101_ = 6.25, P = 0.012) for both sessions (Fig. 3). For omnivore and all species of carabids, the per capita seed consumption was significantly affected by an interaction between the Aphididae biomass and the session, with a negative effect of the Aphididae biomass detected only during the session 1 (Supp. Mat. Table S11). Non-significant effects of the Aphididae biomass were detected for granivore carabids.

The number of seeds eaten per seed-eating (LMM: χ^2^_1,101_ = 27.96, P < 0.001) and per omnivore carabid (LMM: χ^2^_1,101_ = 35.12, P < 0.001) significantly increased with the Collembola biomass during the session 1, but decreased during the session 2 (Fig. 3, Table 2 and Supp. Mat. Table S11). For all carabid species totalled, a negative effect of Collembola biomass on the per capita seed consumption was significant only during the session 2 (Supp. Mat. Table S11). Non-significant effects of the Collembola biomass were detected for granivore carabids. The per capita seed consumption of all carabid groups was never affected by Arachnida prey biomass, irrespective of the sampling session.

The total biomass of alternative prey had a negative significant effect on the per capita seed consumption of all carabid species (LMM: χ^2^_1,101_ = 4.48, P = 0.034), seed-eating carabids (LMM: χ^2^_1,101_ = 7.77, P = 0.005) and omnivore (LMM: χ^2^_1,101_ = 6.37, P = 0.012) in both sessions (Fig. 3, Table 2 and Supp. Mat. Table S11). Non-significant effects of the total biomass of alternative prey were detected for granivore carabids.

## Discussion

Our study suggests that carabids contribute to the predation and regulation of weed seeds in arable fields in European cropland. We show that carabids are negatively correlated with weed seedbank turnover just prior to crop harvest (Table 3), in four countries of Europe, suggesting that weed seed predation by carabids is an important and general natural regulation that works at continental scales, validating our expectation (i). The weed seedbank regulation relationship did not vary with the availability of alternative prey biomass, however, leading us to reject expectation (ii). Our results validate expectation (iii), showing that the AD of carabids was positively related to the predation rates of *P. annua* seeds on seed predation cards, particularly during sampling session 2 (Table 3). The per capita seed consumption of carabids co-varied with the local availability of alternative prey biomass for Aphididae, Collembola and total biomass at the European scale excepted for granivore carabids, corroborating expectation (iv).

**Table 3:** Summary of the results obtained for the four expectations (i, ii, iii and iv). For each of the four groups of carabids studied and according to the session (S1: session 1 and S2: session 2) we indicate whether the relationships tested are significantly positive (green arrow), negative (red arrow) or non-significant (grey arrow).

Expectation			Carabid groups			
			All species	Granivore	Omnivore	Seed-eating
(i)	Effect of carabid AD on the seedbank change		S1: S2:	S1&2:	S1: S2:	S1: S2:
(ii)	Effect of alternative prey on the carabid AD—seedbank change relationship	Aphididae Collembola Arachnida Total	S1&2:	S1&2:	S1&2:	S1&2:
(iii)	Effect of carabid AD on the weed seed predation on seed cards		S1: S2:	S1&2:	S1: S2:	S1: S2:
(iv)	Effect of alternative prey on the per capita seed consumption on seed cards	Aphididae	S1: S2:	S1&2:	S1: S2:	S1&2:
		Collembola	S1: S2:	S1&2:	S1: S2:	S1: S2:
		Arachnida	S1&2:	S1&2:	S1&2:	S1&2:
		Total	S1&2:	S1&2:	S1&2:	S1&2:

### Soil seedbank change and predation of weed seeds from the soil surface by carabids

Our results suggest that when carabids are abundant, the soil seedbank was reduced, validating our expectation (i). This is the first indication of the regulation of the weed seedbank by carabids at continental scales, along a gradient of landscape and farm practice in Europe. Importantly, we found this regulatory effect of carabids on the soil seedbank, for omnivores, seed-eating carabids and when all species of carabids were taken into account. This may suggest that some species currently considered as carnivores in the literature, and therefore in this study, are also consuming seeds in the field, thus contributing to the reduction of the seedbank size (see carnivore section in Supp. Mat. Table S10). These species would typically be considered of lower importance in seed predation studies and our results highlight the current lack of knowledge of the diet of many species^4^ and their contribution to weed regulation. It would further indicate that a revision of the conventional classification of the trophic guilds of carabids may be needed (see Talarico et al.^39^ and references therein), possibly using molecular analysis of the food choice of a large variety of carabid species in differing contexts of prey availability. Based on the outcomes of our analyses, it seems that a large proportion of the carabid community contributes to the seedbank regulation of a wide range of weed species. Carabid species may have complementary foraging strategies^40^ and seed preferences^3,4,6^. We did not detect any effect of the AD of granivores on the soil seedbank change. This is likely to be due to the scarcity of these carabids by comparison with the range of AD observed for the other trophic guilds (Supp. Mat. Table S1). We found indication of the regulation of the seedbank by carabids present in session 2, just prior to harvest. Weed seed shed and dispersal intensifies during the course of the crop growing period to harvest^41^. It seems likely that carabid communities would therefore have the greatest effect in the window just prior the harvest, when weed seeds on the soil surface may have accumulated, rather than earlier in the crop-growing period.

Direct assessment of carabid predation on weed seeds was estimated using predation cards covered with *P. annua* seed that were exposed synchronously with the pitfall traps. These seed predation card results would indicate a pattern of carabids consuming weed seeds at the soil surface that is in agreement with both expectation (iii) and several local-scale studies conducted across Europe^11,14,15^. We find a positive effect of carabid abundance on seed predation along a varied gradient of landscape and farm practice. This relationship was stronger for the granivorous carabids than for the other carabid groups studied, possibly because dominant granivores like *H. affinis* and *H. rufipes* consume substantial quantities of *P. annua* seeds^20,42^, while omnivores such as *P. melanarius* may consume fewer^43^. The positive association between predation rates and AD of omnivorous, seed-eating and all carabids was only significant during session 2 (Table 3), possibly due to changes in the diet of the carabids over time, and to greater consumption of seeds towards the end of the season^41^. These results suggest that all carabid guilds contribute to seed predation in sampling session 2, in contrast to session 1 where only specialist granivores were found to be related to the predation rate on the seed cards. This result is consistent with those on the seedbank regulation, where a signal of regulation was detected only in session 2, when all groups of carabids contributed. Weed seed predation varied between countries, with high levels of predation in the Czech Republic, intermediate levels in Sweden and Austria and low levels in France (Supp. Mat. Fig. S3). Although seed predation cards provide a standardised measurement of seed removal, they have the disadvantage of presenting seeds of a limited number of weed species and therefore only measure predation by carabid species capable of consuming those seed species^15^. *Poa annua* was regularly found to be present in the Czech Republic, Sweden and Austria, but rarely found in the fields sampled in France, possibly explaining the relatively low predation rates there. The seed removal rates varied over time, with higher predation rates during the crop growing season (session 1) than just before harvest (session 2) (Table 2 and Supp. Mat. Fig. S3). This change may reflect co-variation with the composition of carabid communities^44^ and/or switching in their diet during the season^38^ with change in the local availability of animal and plant food resources.

### Local availability of alternative prey modulates seed predation by carabids, but has no detectable effect on the seedbank

The lack of a clear pattern of effect of alternative prey on carabid induced seedbank change may be due to a lack of synchrony between the evaluation of the seedbank, over an entire year, and the sampling of carabids and prey at two sessions within the year. Weed seed regulation occurs all year round^45,46^ and the variation we observe over a year may not be adequately explained by two sample sessions of alternative prey. We therefore also assessed the effect of alternative prey on the seed predation, using predation cards exposed synchronously with the sampling of carabids and prey during the two sessions. Our results on seed cards support expectation (iv) that the effectiveness of carabids to intercept seeds is dependent on the local availability of alternative prey (alternative prey hypothesis). There was a negative effect of the biomass of Aphididae, Collembola and total biomass on the per capita seed consumption for seed-eating carabids, particularly in session 2 (Table 3). These alternative prey groups are readily consumed by the dominant omnivore and granivore species, *P. cupreus, P. melanarius* and *H. rufipes*^25,26^*.* When seeds and alternative prey co-occur in time and space, indirect positive interactions can lead to a negative effect of the availability of alternative prey on weed seed predation, and vice versa, as the foraging carabids switch between different prey types^21^. They may therefore consume more alternative prey than seeds in situations where alternative prey are more abundant or preferred to weed seeds. In places with large quantities of alternative food items, moreover, the encounter rates between carabids and seeds would be reduced and the aggregation of carabids around seed patches would be disturbed^47–49^. High consumption of alternative prey might also lead to carabid satiation^50^ and to the consumption of fewer seeds, including during the periods of seed dispersal just prior to harvest. To date, most of the studies evaluating the effect of alternative prey on predation by carabids have been limited to animal pest prey species in laboratory, and this has generally shown a negative effect of the availability of alternative prey on pest predation^33–35^. Our results also confirm a general negative effect of alternative prey on weed seed predation under field conditions.

We found no evidence of a negative effect of Aphididae biomass on carabid per capita seed consumption during session 2, except for the seed-eating carabids. This result was surprising, given that past molecular analyses sometimes revealed high Aphididae consumption by carabids, specifically in the late in the cropping season^25–27^. Here, the estimate of Aphididae biomass was calculated from vegetation and soil surface sample data and may not, therefore, reflect well prey availability for carabids^51^ estimated from pitfall traps. Moreover, Bilde and Toft^52^ showed that some carabid species, including *P. cupreus,* had low preferences for Aphididae, suggesting that they are lower quality prey than Collembola. Seed predation by granivores do not co-vary with the alternative prey groups, while seed predation by omnivores was affected by Aphididae, Collembola and total biomass. These differences may be a consequence of their diet, with omnivore carabids depending on both seed and animal alternative prey, whereas granivore carabids mainly depend on weed seed resources and occasionally feed on alternative prey.

The negative effects of alternative prey seem to vary with the type of alternative prey present and with the crop-growing period. For example, the negative effect of Aphididae on the seed consumption of omnivores was only observed in session 1 and not in session 2, while the effect of Collembola was positive during the session 1 but negative during the session 2. Such variation may be related to changes in the relative availability of alternative prey to seeds, but it is also possible that there is variation in the diet preferences of the carabids during the crop-growing period. A few laboratory studies have shown evidence of apparent competition, with a negative indirect interaction between prey sharing the same predator, leading to an increase in the predation of a target prey in the presence of alternative prey, including some evidence on carabids eating animal pests^53^. The presence of alternative prey may satisfy the dietary requirements of certain carabid species^54,55^ for a mixed diet, increasing their longevity, fecundity and in-field abundance^30,56^, which could enhanced predation of a targeted prey in the long term. The presence of alternative prey, moreover, might permit rapid field colonisation and a longer temporal persistence of generalist carabids, and allow a rapid exploitation of targeted prey^57^, such as seeds during intense and brief seed rain episodes. This could explain the positive effect of Collembola biomass on the weed seed predation of omnivore and seed-eating carabids during the session 1, or the lack of negative effect of Aphididae during the session 2. Indeed, the aggregation of carabids around Collembola or Aphididae could benefit to seed predation, potentially diluting the negative effects of alternative prey. The positive/negative contribution of alternative prey to predation of weed seeds would differ depending on the period^31^. In early spring, alternative prey could help colonization and maintenance of carabids in the field before weed seed dispersal. Some carabid species, such as *P. cupreus*, would eat alternative prey during the season until weed seeds were available^58^. Carabids would therefore already be present in the fields when weed seeds are dispersed, thus allowing faster seed predation. However, during episodes of weed seed rain, later in the crop growing period, alternative prey would disrupt the predation of the weed seeds.

This correlative study has allowed us to postulate hypotheses for testing, which should guide future research. We expect that: (1) all carabid species contribute to the weed seedbank regulation with efficiencies that depend upon their trophic behaviour; (2) weed seed predation by carabid communities, at the end of the crop-growing period, will play an important role in regulating the seedbank; (3) weed seed predation is modulated by alternative prey, and co-varies with the carabid tropic guild; and, (4) the effect of alternative prey will change throughout the crop-growing period. Future research should test these hypotheses using manipulative experiments, extending the correlative results presented here. This could include the assessment of seedbank change, with and without carabids exclusion treatments, across a range of alternative prey densities over the crop-growing period. Future molecular analyses^25–27,37^ of both alternative and plant prey in carabid gut contents, across these differing contexts, would lead to a better understanding of the role of alternative prey throughout the growing season, and thus to better identify the situations most favourable to weed seed predation and regulation by carabids.

## Materials and methods

### Sampling site and field experimental design

Field experiments were conducted in 60 cereal fields in 2018 in four European countries, with fifteen fields in Austria, the Czech Republic, France and Sweden being selected (Supp. Mat. Fig. S6 and Table S12). Field sizes ranged from 0.55 to 20.73 ha, with an average of 5.58 (SD = 3.84) ha. The fields were selected in order to cover a landscape gradient ranging from 5 to 95% of arable crops in the surrounding 1 km^2^ (Supp. Mat. Table S13). Four fields were grown with winter barley, and 56 fields with winter wheat. Within each field, biological measures were conducted along four transects running from the field margins and extending towards the field centre. There were four sampling points on each transect, at 4, 8, 16 and 32 m from the field border, leading to 16 sampling points per field (Supp. Mat. Fig. S7). Sampling occurred at the ‘mid-season’ of cereal growing (session 1) and ‘just prior to harvest’ (session 2). These periods corresponded to May and June 2018 in France, the Czech Republic and Austria, and to June and July 2018 in Sweden.

### Sampling of carabids

Carabid (Coleoptera: Carabidae) communities were assessed using pitfall traps composed of a plastic beaker (7 cm diameter, > 7 cm depth), half filled by a preservative solution of salt-saturated water. A few drops of odourless soap were added to reduce the surface tension of the solution. The beakers were placed inside PVC sleeves inserted into the soil to allow the replacement of the container without disturbing the surrounding soil. A cover was suspended above each pitfall trap to limit rain inundation. Carabids from the pitfall traps were removed 7 days after trap opening and identified to the species level in the laboratory. The nomenclature followed Löbl & Smetana^59^. Species were then assigned to trophic guilds (carnivore, omnivore, granivore). The main source for the trophic guilds was Homburg et al.^60^, and complementary sources are presented in Supp. Mat. Table S2. Based on the trophic guilds, four groups of carabids were formed for further analysis: all carabid species summed, seed-eating (omnivore + granivore), omnivores and granivores.

### Weed seedbank regulation

We assessed the weed seed ‘regulation’ using the change in the size of the weed seedbank between the time prior sowing in 2017 (initial seedbank) and in 2018 after harvest (follow-up seedbank). Seedbank abundance was estimated by taking 5 soil cores (1.5 L in total between 0–20 cm depth) at the 4 and 32 m sampling points along the transects. The seedbank was estimated by germination of soil samples in a greenhouse under controlled conditions (18/15 °C minimum day/nigh temperature, 12:12 h light:dark cycle). Counting and species identification of the germinated seeds in the samples were done up to 18 weeks after sample preparation. We summed up all the germinated seeds per sample to estimate the initial and follow-up seedbank sizes. Due to a technical problem the seedbank of one Swedish field was removed from the analysis.

### Weed seed predation

Weed seed predation cards^16^, with 50 *Poa annua* seeds, were used to estimate weed seed predation by invertebrates. We selected seeds of *P. annua* because they are preferred by an array of spring breeding carabids^20^ and it is a common weed in Europe. Seeds were glued to 95 × 40 mm card of sand paper (grain size 60). The seed cards were enclosed in a mesh cage (1 cm^2^ wire mesh) to exclude vertebrates. The cards were exposed over 7 days simultaneously with the pitfall traps. The seed predation rate was estimated using the number of seeds removed from seed cards. Combining seed card and carabid data, we derived an estimate of the per capita seed consumption for carabids, calculated as the number of seeds consumed divided by the number of carabids captured per field and session (for each carabid group separately). We used this metric as an estimate of the individual efficiency of carabids for the sole purpose of assessing how it responds to the availability of prey.

### Sampling of alternative prey

Suction sampling was performed on the standing vegetation and on the ground surface to assess the availability of alternative prey using a ‘vortis’ insect suction sampler. Suction sampling was conducted on windless and rainless days when the temperature was above 15 °C. We focused on three animal groups (Arachnida, Aphididae and Collembola) because previous molecular trophic analyses had highlighted that they are prey consumed by carabids^25–27^. We converted the counts of each prey group into a biomass estimate. Arachnida biomass (mg) was estimated using published allometric equations that derive mass from body length^61^. We calculated the average body size of each Arachnida family using the body size of the species captured^62,63^. Aphididae and Collembola were identified at the family and subclass level, respectively. Collembola were converted to counts and biomasses from an abundance index (Supp. Mat. Table S6). The estimated individual masses used for Collembola and Aphididae were 0.082 mg^64^ and 4.5 mg^65^, respectively. Prey counts and biomasses were assessed for each sampling point and each session, except for Austria, for which only the first session was available. We thus had a total of 105 field-sessions.

### Field management and landscape co-variables

In order to control for confounding co-variates, which potentially can affect weed seedbank and seed predation, we decided to include an estimation of pesticide management intensity and land use differences in the landscape as co-variables in our analyses. All fields were managed with the use of herbicides and insecticides on demand, except in Austria where no or few pesticide were used. As a proxy of field pesticide management intensity, we used the number of field visits by the farmer to spray pesticide during all the crop-growing period (Supp. Mat. Table S13). This information could not be collected for two fields in Sweden, leading to estimation for 57 fields in total. For land use differences we used the proportion of arable crops in the surrounding 1 km^2^.

### Statistical analysis

#### Final dataset description

For analyses, all samples from each field for the two sessions were summed. This permitted us to reduce the influence of outliers and natural, stochastic processes attributable to fine-scale microclimatic conditions. Some pitfall traps were destroyed in 7 fields (2 and 5 fields during session 1 and 2, respectively). In order to compare these fields with the others, we standardized the number of carabids captured prior to conducting statistical analyses. We considered that the AD of carabids was proportional to the number of pitfall traps, and we extrapolated captures for 16 traps per field following the method of Bohan *et al.* and Brooks *et* *al.*^66,67^.

#### Models of expectation (Supp. Mat. Fig. S9)

#### (i) The relationship between the weed seedbank change and the AD of carabids

We investigated the effect of the log-transformed AD of carabids and the log-transformed initial seedbank on the log-transformed follow-up seedbank using linear modelling (LM).

#### (ii) Weed seedbank regulation relationship will co-vary with the availability of alternative prey biomass

We tested the effect of the initial seedbank and the interaction between the AD of carabids and the biomass of alternative prey on the follow-up seedbank using LM. All variables were log-transformed.

#### (iii) The relationship between the AD of carabids and weed seed predation

We tested the effect of the log-transformed AD of carabids in interaction with the session on the weed seed predation rates on the seed cards using Generalised Linear Mixed Model (GLMM), with the binomial (link = “logit”) family and expressed predation rates as the proportion of seed removed on the seed cards.

#### (iv) The local availability of alternative prey biomass will be a significant co-variate of the per capita seed consumption by carabids

We tested the effect of the log-transformed biomass of alternative prey in interaction with the session on the per capita seed consumption of carabids using Linear Mixed Models (LMM). We used the per capita seed consumption rather than seed predation in this analysis because we want to see how the effectiveness of carabids in consuming seeds depends on the alternative prey biomass.

For (i) and (ii), separate models were fitted for each session, as we faced convergence problems when including both sessions in the same model, therefore no random effects were necessary. For (iii) and (iv), because the session factor was included in models we also included the Field ID as a random effect to account for pseudo-replication within a field. For all models the proportion of crops (pCrop) and the pesticide intensity were included as fixed co-variables to account for environmental differences between countries. The combination of these two co-variables provides an estimate of the environmental differences between the four countries. In order to avoid model overfitting, and since these two co-variables allow for a description of the differences between countries (Supp. Mat. Figure S8), we did not include the country effect in models.

For all the four steps, separate models were fitted for each carabid group. For the steps (ii) and (iv), separate models were also fitted for each prey group. The goodness-of-fit of the models was assessed by checking normality and randomness of residuals. To improve normality and homoscedasticity of residuals, the per capita seed consumption, the seedbanks, carabid AD and prey biomass were natural logarithm transformed. We verified the absence of multicollinearity between explanatory variables using Variation Inflation Factors (VIFs). All VIFs for non interaction effects were bellow 3, indicating low collinearity. P values were interpreted using a type = “III” when interaction terms were significant, otherwise using type = “II” analysis-of-variance tables for GLMMs and LM(M)s with Wald chi-square or F tests, respectively, using the ‘Anova’ procedure ( package ‘car’^68^). Where interaction terms were significant, we assessed whether the slopes were significantly different from 0 for the two sessions, using the procedure ‘sim_slopes’ ( package ‘jtools’^69^). Where significant, we used estimated marginal means to compare the different sessions with pairwise comparison using the procedure ‘emmeans’ (package ‘emmeans’^70^). Partial residuals were used in the seedbank change graphs because they provide a better estimation of a statistically adjusted response variables when more than one factor in the regression model was significant. All figures are created using the procedure ‘predictorEffect’ (package ‘effects’^68^). We tested for correlation between carabid and prey biomass using LMM with session, landscape and management co-variables as a fixed effect and Field as a random effect (Supp. Mat. Table S14). Models were performed using the procedures ‘glmer’, ‘lmer’ and ‘lm’, respectively, implemented in the package ‘lme4′^71^ and ‘stats’. All analyses and figures were conducted in R version 3.6.1^72^.

## Supplementary information

Supplementary Information.

## Data Availability

The datasets analysed during the current study are available from the corresponding author on request.
