# Supplementary material for: The resilience of weed seedbank regulation by carabid beetles, at continental scales, to alternative prey
Source: Sci Rep. 2020 Nov 9;10:19315. doi: 10.1038/s41598-020-76305-w (PMC7652833; doi:10.1038/s41598-020-76305-w)
Supplement: Supplementary file 1 — Supplementary Information. [file 41598_2020_76305_MOESM1_ESM.pdf]

Supplementary materials

Title: The resilience of weed seedbank regulation by carabid beetles, at continental scales, to alternative prey.

Benjamin Carbonne[1*], Sandrine Petit[1], Veronika Neidel[2], Hana Foffova[3,4], Eirini Daouti[5], Britta Frei[1,2], Jiří Skuhrovec[3], Milan Řezáč[3], Pavel Saska[3], CorinnaWallinger[2], Michael Traugott[2] & David A. Bohan[1]

[1]Agroécologie, AgroSup Dijon, INRAE, Université de Bourgogne Franche-Comté, F-21000 Dijon, France

[2]Mountain Agriculture Research Unit, Institute of Ecology, University of Innsbruck, Innsbruck, Austria

[3]Functional Diversity in Agro-Ecosystems, Crop Research Institute, Drnovská 507, 161 06 Praha6 – Ruzyně, Czech Republic

[4]Department of Ecology, Faculty of Environmental Sciences, Czech University of Life Sciences Prague, Kamýcká 129, 165 00 Praha– Suchdol, Czech Republic

[5]Department of Ecology, Swedish University of Agricultural Sciences, Box 7044, SE-75007 Uppsala, Sweden

*Corresponding author: Benjamin Carbonne[1]
Email address: benjamin_carbonne@hotmail.fr
Postal address: 17 rue Sully, BP 86510, 21065 Dijon cedex, France

Supplementary material summary:

List of abbreviations: ... 2
Description of carabid catches: ... 2
Seedbank description: ... 8
Seed card description: ... 9
Description of alternative prey : ... 10
Effect of the different groups of carabids on the weed seedbank change and modulation by the alternative prey ... 14
Effect of the different group of carabids on the weed seed predation (seed card), and effect of the biomass of alternative prey on the per capita seed consumption ... 17
Maps and sampling design: ... 19
Correlation between the different group of carabids and the biomass of the different alternative prey groups ... 21
References ... 23

List of abbreviations:

AU: Austria
CZ: The Czech Republic
FR: France
SW: Sweden

Description of carabid catches:

Supp. Mat. Table S1. Descriptive statistics (mean (SD), median [min, max] and sum) for activity-density per field per session of the carabids caught sorted by trophic guilds and countries. In case of missing traps on the fields, i.e. less than 16 traps per field, the activity-density obtained was extrapolated for 16 traps. All countries included 15 fields with two sample sessions (n = 30).

	AU (n=30)	CZ (n=30)	FR (n=30)	SW (n=30)	Overall (n=120)
**AD seed-eating**					
Mean (SD)	724 (591)	250 (179)	157 (132)	451 (276)	395 (404)
Median [Min, Max]	501 [146, 2470]	207 [38.0, 744]	106 [18.0, 628]	349 [80.0, 1190]	283 [18.0, 2470]
Sum	21708	7488	4715	13521	47433
**AD granivore**					
Mean (SD)	70.4 (101)	26.7 (26.6)	11.9 (16.8)	58.8 (52.0)	41.9 (63.0)
Median [Min, Max]	28.5 [1.00, 441]	13.0 [1.00, 101]	5.00 [0.00, 64.0]	42.5 [5.00, 185]	17.5 [0.00, 441]
Sum	2111	801	357	1763	5032
**AD omnivore**					
Mean (SD)	653 (522)	223 (172)	145 (122)	392 (280)	353 (367)
Median [Min, Max]	492 [13.0, 2130]	195 [26.0, 694]	103 [16.0, 580]	332 [61.0, 1090]	247 [13.0, 2130]
Sum	19598	6687	4358	11758	42401
**AD all carabid**					
Mean (SD)	985 (647)	432 (313)	383 (293)	570 (374)	593 (487)
Median [Min, Max]	754 [187, 2850]	306 [69.0, 1280]	277 [86.0, 1260]	429 [84.0, 1380]	439 [69.0, 2850]
Sum	29559	12965	11479	17101	71104
**Richness**					
Mean (SD)	19.1 (3.44)	14.9 (5.18)	16 (5.03)	15 (3.69)	15.8 (4.69)
Median [Min, Max]	20.0 [14.0, 25.0]	14.0 [6.00, 30.0]	14.0 [9.00, 29.0]	14.5 [8.00, 23.0]	15.0 [6.00, 30.0]
Total richness	43	70	64	50	117

Supp. Mat. Figure S1. Boxplot presenting the activity-density (AD) per field of seed-eating (omnivores + granivores), omnivores, granivores and of all carabid species for the 60 fields with colours indicating the session 1 and 2. We distinguished the four countries: AU, CZ, FR and SW. The plot is created using R version 3.6.1[1] and the package 'ggplot2'[2].

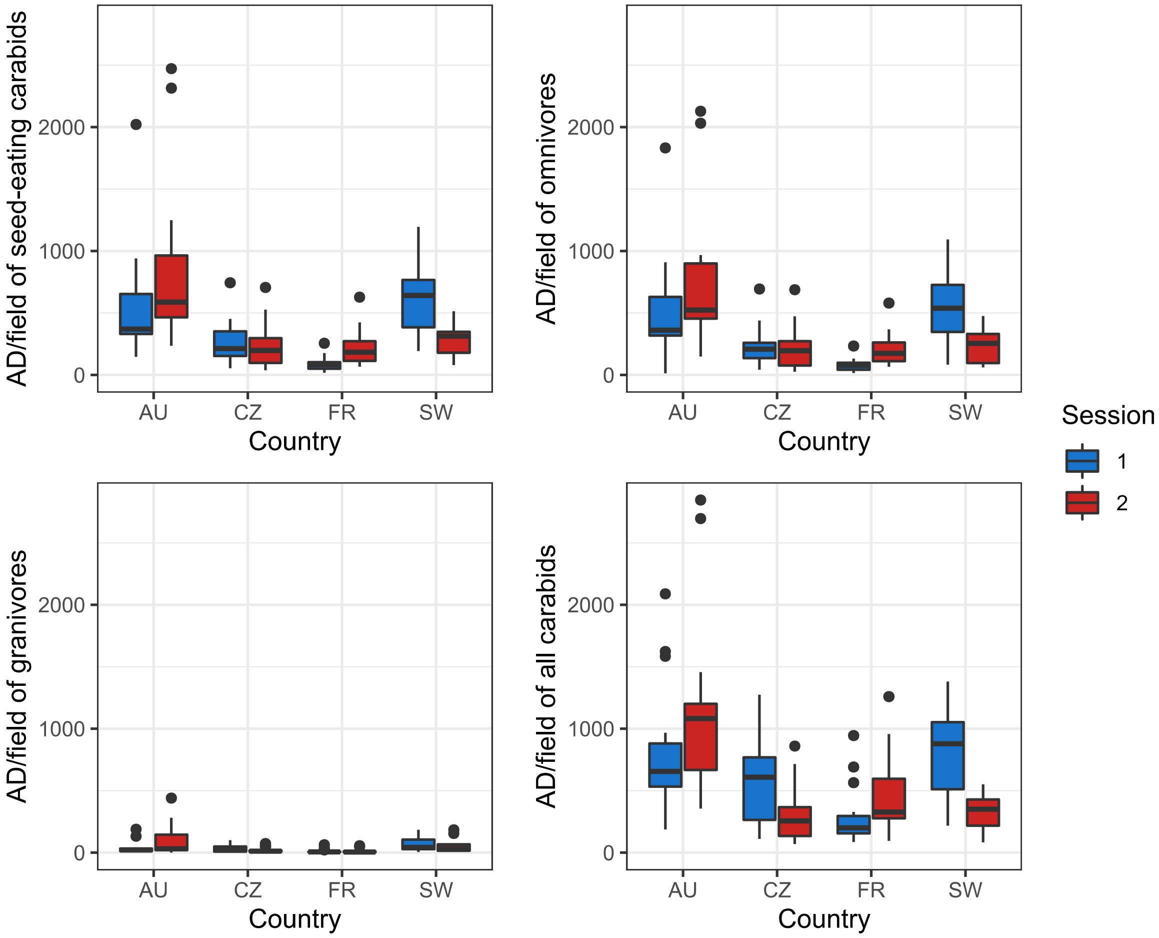

Supp. Mat. Table S2. Description of the 117 carabids species trapped in experiment (15 fields and 2 sessions for each 4 countries, leading to 120 field-session). The main source for trophic guilds is Homburg et al., (2014)[3], and complementary sources: Fournier et al. (2015)[4]; Purtauf et al. (2005)[5]; Saska et al. (2008, 2019)[6,7]; Honek et al. (2003, 2007)[8,9]; Koprdova et al. (2008)[10]. Trophic guilds were indicated with "C:" Carnivorous; "O:" Omnivorous and "G:" Granivorous. Distribution details the total captured (number of individuals and the corresponding percentage), the number of carabid captured per field and session (mean (SD) and median [min, max]), field occurrence (number of fields-session with presence and the corresponding percentage) and country with presence.

				Species abundance			***Field-session species occurrence***	***Country with presence***
Code	**Species**	**Authority**	**Trophic guild**	**Total (%)**	**AD/field and session** **Mean (SD)**	**Median [Min; Max]**	**Number (%)**	
ABAPLL	*Abax parallelus*	(Duftschmid, 1812)	C	9 (0.01%)	0.08 (0.49)	1 [1; 2]	7 (5.83%)	AU;CZ;FR
ABAPLP	*Abax parallelepipedus*	(Piller & Mitterpacher, 1783)	C	19 (0.03%)	0.16 (1.06)	2.5 [1; 4]	8 (6.67%)	AU;CZ
ACUMER	*Acupalpus meridianus*	(Linnaeus, 1760)	O	4 (0.01%)	0.03 (0)	1 [1; 1]	4 (3.33%)	CZ;FR
AGODUF	*Agonum duftschmidi*	Schmidt, 1994	C	1 (0%)	0.01 (NA)	1 [1; 1]	1 (0.83%)	CZ
AGOMUE	*Agonum muelleri*	(Herbst, 1784)	O	4702 (6.67%)	39.18 (97.15)	68.5 [1; 499]	58 (48.33%)	AU;FR;SW
AGONIG	*Agonum nigrum*	Dejean, 1828	C	11 (0.02%)	0.09 (0.79)	1 [1; 3]	7 (5.83%)	FR
AGOSEX	*Agonum sexpunctatum*	(Linnaeus, 1758)	C	463 (0.66%)	3.86 (18.92)	11 [1; 96]	30 (25%)	AU
AGOTHO	*Agonum thoreyi*	Dejean, 1828	C	1 (0%)	0.01 (NA)	1 [1; 1]	1 (0.83%)	SW
AGOVIR	*Agonum viridicupreum*	(Goeze, 1777)	C	2 (0%)	0.02 (0)	1 [1; 1]	2 (1.67%)	FR
AMAAEN	*Amara aenea*	(De Geer, 1774)	H	576 (0.82%)	4.8 (26.7)	4.5 [1; 96]	32 (26.67%)	AU;CZ;SW
AMAAUL	*Amara aulica*	(Panzer, 1797)	H	1 (0%)	0.01 (NA)	1 [1; 1]	1 (0.83%)	CZ
AMABIF	*Amara bifrons*	(Gyllenhal, 1810)	H	8 (0.01%)	0.07 (0)	4 [4; 4]	2 (1.67%)	SW
AMACOM	*Amara communis*	(Panzer, 1797)	H	9 (0.01%)	0.08 (0.84)	2 [1; 3]	5 (4.17%)	AU;SW
AMACON	*Amara consularis*	(Duftschmid, 1812)	H	25 (0.04%)	0.21 (9.84)	1.5 [1; 21]	4 (3.33%)	AU;FR
AMAFAM	*Amara familiaris*	(Duftschmid, 1812)	H	49 (0.07%)	0.41 (4.82)	1 [1; 21]	17 (14.17%)	AU;CZ;SW
AMAFUL	*Amara fulva*	(O.F. Müller, 1776)	H	4 (0.01%)	0.03 (NA)	4 [4; 4]	1 (0.83%)	SW
AMALIT	*Amara littorea*	Thomson, 1857	H	12 (0.02%)	0.1 (1.26)	1.5 [1; 4]	6 (5%)	CZ
AMALUN	*Amara lunicollis*	Schiødte, 1837	H	1 (0%)	0.01 (NA)	1 [1; 1]	1 (0.83%)	CZ
AMAOVA	*Amara ovata*	(Fabricius, 1792)	H	15 (0.02%)	0.13 (1.66)	1 [1; 6]	9 (7.5%)	AU;CZ
AMAPLE	*Amara plebeja*	(Gyllenhal, 1810)	H	107 (0.15%)	0.89 (9.51)	1 [1; 31]	17 (14.17%)	AU;CZ;SW
AMASIM	*Amara similata*	(Gyllenhal, 1810)	H	653 (0.93%)	5.44 (49.85)	1.5 [1; 244]	34 (28.33%)	AU;CZ;FR;SW

ANCDOR	*Anchomenus dorsalis*	(Pontoppidan, 1763)	C	10501 (14.89%)	87.51 (116.36)	54 [1; 585]	112 (93.33%)	AU;CZ;FR;SW
ANIBIN	*Anisodactylus binotatus*	(Fabricius, 1787)	H	136 (0.19%)	1.13 (7.32)	4 [1; 23]	19 (15.83%)	AU
ANISIG	*Anisodactylus signatus*	Panzer, 1797	H	5 (0.01%)	0.04 (0.58)	2 [1; 2]	3 (2.5%)	AU;CZ;FR
ASAFLA	*Asaphidion flavipes*	(Linnaeus, 1760)	C	15 (0.02%)	0.13 (0.5)	1 [1; 2]	11 (9.17%)	AU;FR;SW
BADBUL	*Badister bullatus*	(Schrank, 1798)	C	11 (0.02%)	0.09 (0.32)	1 [1; 2]	10 (8.33%)	CZ;FR;SW
BADCOL	*Badister collaris*	Motschulsky, 1844	C	1 (0%)	0.01 (NA)	1 [1; 1]	1 (0.83%)	CZ
BADSOD	*Badister sodalis*	(Duftschmid, 1812)	C	19 (0.03%)	0.16 (0.78)	1 [1; 3]	13 (10.83%)	AU;FR
BADUNI	*Badister unipustulatus*	Bonelli, 1813	C	1 (0%)	0.01 (NA)	1 [1; 1]	1 (0.83%)	FR
BEMAEN	*Bembidion aeneum*	Germar, 1823	C	63 (0.09%)	0.53 (6.78)	3.5 [1; 19]	10 (8.33%)	SW
BEMASS	*Bembidion assimile*	Gyllenhal, 1810	C	1 (0%)	0.01 (NA)	1 [1; 1]	1 (0.83%)	CZ
BEMGUT	*Bembidion guttula*	(Fabricius, 1792)	C	3 (0%)	0.03 (0.71)	1.5 [1; 2]	2 (1.67%)	SW
BEMLAM	*Bembidion lampros*	(Herbst, 1784)	C	1345 (1.91%)	11.21 (22.03)	8 [1; 141]	87 (72.5%)	AU;CZ;FR;SW
BEMLAT	*Bembidion latinum*	Netolitzky, 1911	C	1 (0%)	0.01 (NA)	1 [1; 1]	1 (0.83%)	FR
BEMLUN	*Bembidion lunulatum*	(Geoffroy, 1785)	C	8 (0.01%)	0.07 (0)	1 [1; 1]	8 (6.67%)	FR
BEMOBT	*Bembidion obtusum*	Audinet-Serville , 1821	C	173 (0.25%)	1.44 (7.42)	2 [1; 45]	38 (31.67%)	CZ;FR;SW
BEMPRO	*Bembidion properans*	(Stephens, 1828)	C	1195 (1.69%)	9.96 (28.2)	15 [1; 121]	48 (40%)	AU;CZ;FR
BEMQUA	*Bembidion quadrimaculatum*	(Linnaeus, 1760)	C	66 (0.09%)	0.55 (4.95)	1 [1; 24]	22 (18.33%)	AU;CZ;FR;SW
BEMTET	*Bembidion tetracolum*	Say, 1823	C	593 (0.84%)	4.94 (116.87)	2 [1; 484]	17 (14.17%)	AU;SW
BLEDIS	*Blemus discus*	(Fabricius, 1792)	C	6 (0.01%)	0.05 (0.58)	1.5 [1; 2]	4 (3.33%)	CZ;SW
BRACRE	*Brachinus crepitans*	(Linnaeus, 1758)	C	1215 (1.72%)	10.13 (95.39)	11.5 [1; 470]	30 (25%)	CZ;FR
BRAELE	*Brachinus elegans*	Chaudoir, 1842	C	8 (0.01%)	0.07 (0.89)	1 [1; 3]	5 (4.17%)	FR
BRAEXP	*Brachinus explodens*	Duftschmid, 1812	C	847 (1.2%)	7.06 (223.2)	3.5 [1; 779]	12 (10%)	CZ
BRASCL	*Brachinus sclopeta*	(Fabricius, 1792)	C	127 (0.18%)	1.06 (16.86)	2 [1; 68]	17 (14.17%)	FR
BROCEP	*Broscus cephalotes*	(Linnaeus, 1758)	C	21 (0.03%)	0.18 (7.78)	10.5 [5; 16]	2 (1.67%)	AU
CALFUS	*Calathus fuscipes*	(Goeze, 1777)	O	306 (0.43%)	2.55 (8.76)	2 [1; 45]	55 (45.83%)	AU;CZ;SW
CALLUN	*Callistus lunatus*	(Fabricius, 1775)	C	1 (0%)	0.01 (NA)	1 [1; 1]	1 (0.83%)	FR
CALMEL	*Calathus melanocephalus*	(Linnaeus, 1758)	C	26 (0.04%)	0.22 (6.34)	1 [1; 18]	7 (5.83%)	AU;CZ;FR;SW
CARARN	*Carabus auronitens*	Fabricius, 1792	C	4 (0.01%)	0.03 (0.58)	1 [1; 2]	3 (2.5%)	AU;FR
CARART	*Carabus auratus*	Linnaeus, 1760	C	13 (0.02%)	0.11 (2.51)	2 [1; 7]	5 (4.17%)	FR
CARCAN	*Carabus cancellatus*	Illiger, 1798	C	23 (0.03%)	0.19 (4.35)	2 [1; 13]	7 (5.83%)	AU;CZ
CARCON	*Carabus convexus*	Fabricius, 1775	C	4 (0.01%)	0.03 (0.58)	1 [1; 2]	3 (2.5%)	CZ
CARCOR	*Carabus coriaceus*	Linnaeus, 1758	C	11 (0.02%)	0.09 (1.6)	1 [1; 5]	6 (5%)	CZ;FR;SW
CARGRA	*Carabus granulatus*	Linnaeus, 1758	C	984 (1.39%)	8.2 (19.49)	10 [1; 82]	57 (47.5%)	AU;CZ;SW
CARHOR	*Carabus hortensis*	Linnaeus, 1758	C	2 (0%)	0.02 (0)	1 [1; 1]	2 (1.67%)	CZ

CARMON	*Carabus monilis*	Fabricius, 1792	C	1 (0%)	0.01 (NA)	1 [1; 1]	1 (0.83%)	FR
CARNEM	*Carabus nemoralis*	O. F. Müller, 1764	C	9 (0.01%)	0.08 (0.35)	1 [1; 2]	8 (6.67%)	AU;FR;SW
CHLNIG	*Chlaenius nigricornis*	(Fabricius, 1787)	C	8 (0.01%)	0.07 (0.52)	1 [1; 2]	6 (5%)	CZ;FR;SW
CLIFOS	*Clivina fossor*	(Linnaeus, 1758)	C	227 (0.32%)	1.89 (6.01)	4 [1; 21]	37 (30.83%)	AU;CZ;FR;SW
CRYMEL	*Cryptophonus melancholicus*	(Dejean, 1829)	H	2 (0%)	0.02 (0)	1 [1; 1]	2 (1.67%)	AU
DEMATR	*Demetrias atricapillus*	(Linnaeus, 1758)	C	21 (0.03%)	0.18 (0.85)	1 [1; 4]	14 (11.67%)	CZ;FR;SW
DIAGER	*Diachromus germanus*	(Linnaeus, 1758)	H	3 (0%)	0.03 (NA)	3 [3; 3]	1 (0.83%)	FR
DOLHAL	*Dolichus halensis*	(Schaller, 1783)	C	22 (0.03%)	0.18 (6.4)	3 [1; 15]	4 (3.33%)	AU;CZ;SW
DYSGLO	*Dyschirius globosus*	(Herbst, 1784)	C	235 (0.33%)	1.96 (17.13)	7 [1; 59]	16 (13.33%)	AU
HARAFF	*Harpalus affinis*	(Schrank, 1781)	H	998 (1.41%)	8.32 (13.75)	5 [1; 77]	85 (70.83%)	AU;CZ;FR;SW
HARATR	*Harpalus atratus*	Latreille, 1804	H	5 (0.01%)	0.04 (0)	1 [1; 1]	5 (4.17%)	CZ;FR
HARDIM	*Harpalus dimidiatus*	(Rossi, 1790)	H	28 (0.04%)	0.23 (1.52)	2 [1; 6]	13 (10.83%)	FR
HARDIS	*Harpalus distinguendus*	(Duftschmid, 1812)	H	61 (0.09%)	0.51 (5.53)	1 [1; 18]	16 (13.33%)	AU;CZ;FR;SW
HARHON	*Harpalus honestus*	(Duftschmid, 1812)	H	5 (0.01%)	0.04 (0.5)	1 [1; 2]	4 (3.33%)	CZ;FR
HARLAT	*Harpalus latus*	(Linnaeus, 1758)	H	2 (0%)	0.02 (0)	1 [1; 1]	2 (1.67%)	CZ
HARLUT	*Harpalus luteicornis*	(Duftschmid, 1812)	H	11 (0.02%)	0.09 (0.44)	1 [1; 2]	9 (7.5%)	CZ
HARRUB	*Harpalus rubripes*	Duftschmid, 1812	H	3 (0%)	0.03 (0)	1 [1; 1]	3 (2.5%)	FR
HARRUF	*Harpalus rufipes*	(DeGeer, 1774)	H	2140 (3.03%)	17.83 (30.3)	9 [1; 156]	105 (87.5%)	AU;CZ;FR;SW
HARSIG	*Harpalus signaticornis*	(Duftschmid, 1812)	H	20 (0.03%)	0.17 (1.89)	1 [1; 6]	10 (8.33%)	CZ;FR
HARSMA	*Harpalus smaragdinus*	(Duftschmid, 1812)	H	1 (0%)	0.01 (NA)	1 [1; 1]	1 (0.83%)	FR
HARTAR	*Harpalus tardus*	(Panzer, 1796)	H	17 (0.02%)	0.14 (0.93)	1 [1; 4]	11 (9.17%)	AU;CZ;FR;SW
LEIFER	*Leistus ferrugineus*	(Linnaeus, 1758)	C	3 (0%)	0.03 (0)	1 [1; 1]	3 (2.5%)	CZ
LEIFUL	*Leistus fulvibarbis*	Dejean, 1826	C	2 (0%)	0.02 (0)	1 [1; 1]	2 (1.67%)	FR
LORPIL	*Loricera pilicornis*	(Fabricius, 1775)	C	245 (0.35%)	2.04 (5.06)	2 [1; 22]	57 (47.5%)	AU;CZ;FR;SW
MASWET	*Masoreus wetterhalli*	(Gyllenhal, 1813)	O	1 (0%)	0.01 (NA)	1 [1; 1]	1 (0.83%)	SW
MICMIN	*Microlestes minutulus*	(Goeze, 1777)	C	9 (0.01%)	0.08 (3.46)	1 [1; 7]	3 (2.5%)	CZ;SW
MICSP	*Microlestes* sp.		C	13 (0.02%)	0.11 (0.74)	1.5 [1; 3]	8 (6.67%)	FR
MOLELA	*Molops elatus*	(Fabricius, 1801)	C	1 (0%)	0.01 (NA)	1 [1; 1]	1 (0.83%)	CZ
NEBBRE	*Nebria brevicollis*	(Fabricius, 1792)	C	137 (0.19%)	1.14 (3.46)	2 [1; 16]	40 (33.33%)	AU;CZ;FR;SW
NEBSAL	*Nebria salina*	Fairmaire & Laboulbène, 1854	C	96 (0.14%)	0.8 (12.86)	2.5 [1; 46]	12 (10%)	FR
NOTAES	*Notiophilus aestuans*	Dejean, 1826	C	2 (0%)	0.02 (0)	1 [1; 1]	2 (1.67%)	FR;SW
NOTAQU	*Notiophilus aquaticus*	(Linnaeus, 1758)	C	5 (0.01%)	0.04 (0)	1 [1; 1]	5 (4.17%)	CZ;SW
NOTBIG	*Notiophilus biguttatus*	(Fabricius, 1779)	C	21 (0.03%)	0.18 (1.41)	2 [1; 5]	9 (7.5%)	CZ;FR

NOTPAL	*Notiophilus palustris*	(Duftschmid, 1812)	C	18 (0.03%)	0.15 (0.77)	1 [1; 3]	13 (10.83%)	AU;CZ;FR;SW
NOTQUA	*Notiophilus quadripunctatus*	(Dejean, 1826)	C	17 (0.02%)	0.14 (0.63)	1 [1; 3]	13 (10.83%)	FR
OPHAZU	*Ophonus azureus*	(Fabricius, 1775)	H	28 (0.04%)	0.23 (2.86)	1.5 [1; 9]	10 (8.33%)	AU;CZ;FR
OPHSP	*Ophonus* (*Metophonus*) sp.		H	1 (0%)	0.01 (NA)	1 [1; 1]	1 (0.83%)	CZ
OXYOBS	*Oxypselaphus obscurus*	(Herbst, 1784)	C	6 (0.01%)	0.05 (0)	3 [3; 3]	2 (1.67%)	CZ
PANBIP	*Panagaeus bipustulatus*	(Fabricius, 1775)	C	1 (0%)	0.01 (NA)	1 [1; 1]	1 (0.83%)	CZ
PATATR	*Patrobus atrorufus*	(Strøm, 1768)	C	30 (0.04%)	0.25 (8.37)	1.5 [1; 22]	6 (5%)	SW
PLAASS	*Platynus assimilis*	(Paykull, 1790)	C	23 (0.03%)	0.19 (1.3)	2 [1; 5]	11 (9.17%)	AU;CZ;FR
POECUP	*Poecilus cupreus*	(Linnaeus, 1758)	O	17105 (24.25%)	142.54 (267.77)	54 [1; 1592]	117 (97.5%)	AU;CZ;FR;SW
POELEP	*Poecilus lepidus*	(Leske, 1785)	C	9 (0.01%)	0.08 (0.84)	1 [1; 3]	6 (5%)	AU
POEVER	*Poecilus versicolor*	(Sturm, 1824)	C	3355 (4.76%)	27.96 (151.87)	20.5 [1; 653]	38 (31.67%)	AU;CZ;SW
PTEANT	*Pterostichus anthracinus*	(Illiger, 1798)	C	326 (0.46%)	2.72 (51.22)	20.5 [1; 128]	8 (6.67%)	AU
PTEMAD	*Pterostichus madidus*	(Fabricius, 1775)	O	1 (0%)	0.01 (NA)	1 [1; 1]	1 (0.83%)	FR
PTEMEL	*Pterostichus melas*	(Creutzer, 1799)	C	37 (0.05%)	0.31 (11.9)	4 [2; 27]	4 (3.33%)	CZ
PTEMLR	*Pterostichus melanarius*	(Illiger, 1798)	O	17183 (24.36%)	143.19 (181.95)	76.5 [1; 807]	112 (93.33%)	AU;CZ;FR;SW
PTENIG	*Pterostichus niger*	(Schaller, 1783)	C	691 (0.98%)	5.76 (26.88)	5.5 [1; 108]	38 (31.67%)	AU;CZ;SW
PTEOBL	*Pterostichus oblongopunctatus*	(Fabricius, 1787)	C	5 (0.01%)	0.04 (0.71)	2.5 [2; 3]	2 (1.67%)	AU
PTESTR	*Pterostichus strenuus*	(Panzer, 1797)	C	20 (0.03%)	0.17 (4.76)	1.5 [1; 13]	6 (5%)	CZ
PTEVER	*Pterostichus vernalis*	(Panzer, 1796)	C	131 (0.19%)	1.09 (4.94)	2 [1; 19]	33 (27.5%)	AU;CZ;FR;SW
SCYOBL	*Scybalicus oblongiusculus*	(Dejean, 1829)	H	4 (0.01%)	0.03 (1.41)	2 [1; 3]	2 (1.67%)	FR
STOPUM	*Stomis pumicatus*	(Panzer, 1796)	C	17 (0.02%)	0.14 (2.07)	2 [1; 7]	7 (5.83%)	CZ;FR;SW
SYNOBS	*Syntomus obscuroguttatus*	(Duftschmid, 1812)	C	37 (0.05%)	0.31 (3.63)	1.5 [1; 12]	12 (10%)	FR
SYNSP	*Syntomus* sp.		C	1 (0%)	0.01 (NA)	1 [1; 1]	1 (0.83%)	FR
SYNTRU	*Syntomus truncatellus*	(Linnaeus, 1760)	C	7 (0.01%)	0.06 (1.15)	3 [1; 3]	3 (2.5%)	CZ;SW
SYNVIV	*Synuchus vivalis*	(Illiger, 1798)	O	39 (0.06%)	0.33 (2.37)	1 [1; 9]	17 (14.17%)	AU;CZ;SW
TACBIS	*Tachys bistriatus*	(Duftschmid, 1812)	C	1 (0%)	0.01 (NA)	1 [1; 1]	1 (0.83%)	FR
TREMIC	*Trechoblemus micros*	(Herbst, 1784)	C	1 (0%)	0.01 (NA)	1 [1; 1]	1 (0.83%)	CZ
TREQUA	*Trechus quadristriatus*	(Schrank, 1781)	O	2562 (3.63%)	21.35 (54.16)	11 [1; 271]	79 (65.83%)	AU;CZ;FR;SW
TRESEC	*Trechus secalis*	(Paykull, 1790)	O	108 (0.15%)	0.9 (9.14)	2 [1; 33]	17 (14.17%)	CZ;SW
All carabid species				70545 (100%)	587.88 (104.3)	424.5 [0; 1592]	120 (100%)	AU;CZ;FR;SW

Supp. Mat. Figure S2. The ten most abundant carabid species for each country-session. Colours represent the different trophic guilds, with red for carnivorous, orange for omnivorous and green for granivorous species. In case of missing traps on the fields, i.e. less than 16 traps per field, the activity-density obtained was extrapolated for 16 traps. The plot is created using R version 3.6.1[1] and the package ‘ggplot2’[2].

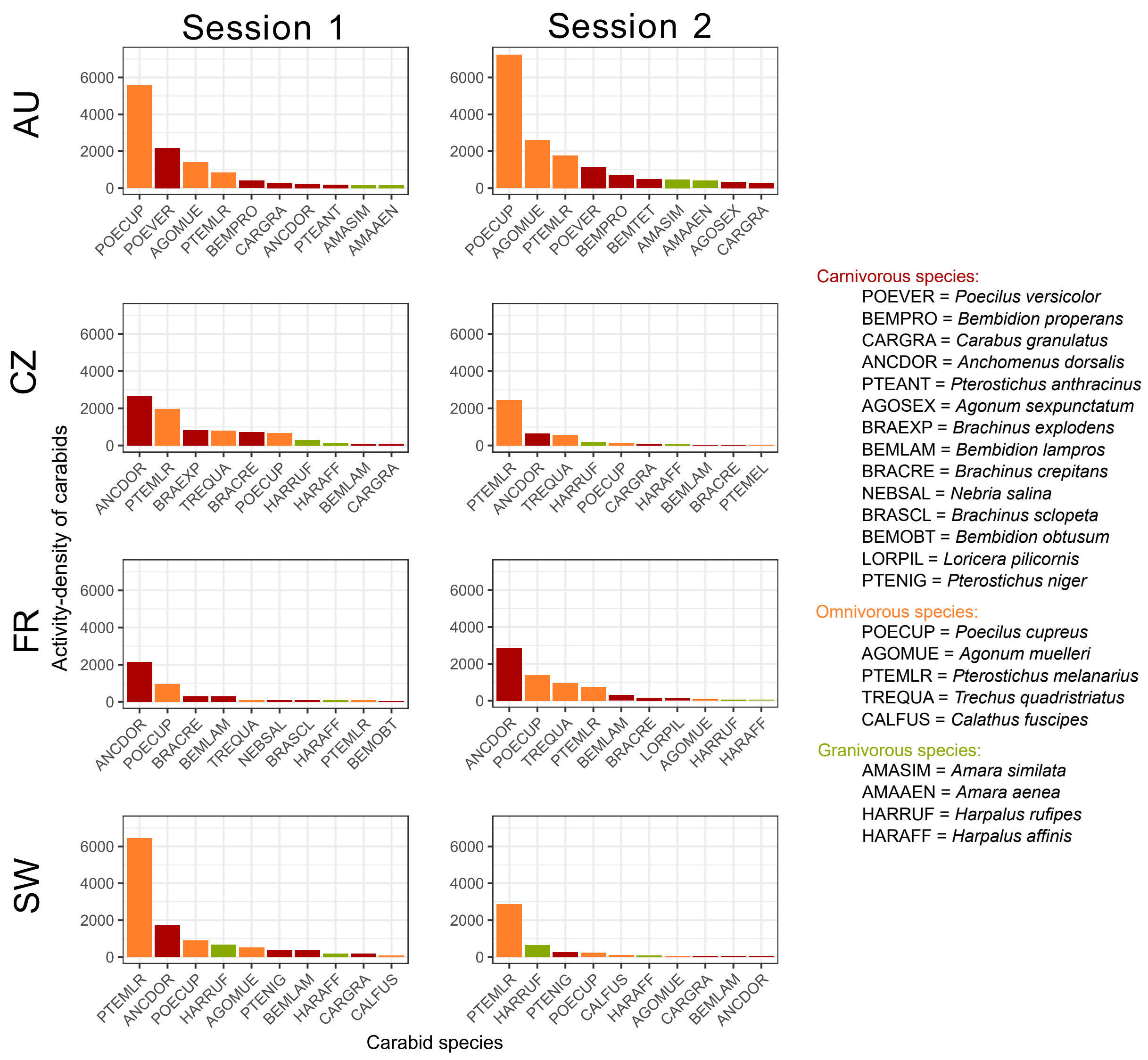

Seedbank description:

Supp. Mat. Table S3. Descriptive statistics (mean (SD), median [min, max], and total sum (also express as percentage)) for the initial and the follow-up seedbank estimation before and after the crop cultivation, and for the seedbank change calculated as -log(follow-up seedbank / initial seedbank) according to the country.

	AU (n=15)	CZ (n=30)	FR (n=30)	SW (n=28)	Overall (n=103)
**Initial seedbank**					
Mean (SD)	228 (157)	142 (84.0)	36.5 (43.0)	201 (171)	140 (138)
Median [Min, Max]	187 [69.0, 574]	113 [35.0, 338]	24.0 [3.00, 181]	119 [52.0, 637]	94.0 [3.00, 637]
Total sum (%)	3427 (23.8%)	4246 (29.6%)	1094 (7.6%)	5614 (39.0%)	14381 (100%)
**Follow-up seedbank**					
Mean (SD)	162 (131)	189 (86.4)	38.4 (25.2)	181 (138)	139 (118)

Median [Min, Max]	118 [36.0, 528]	199 [59.0, 332]	34.0 [7.00, 106]	149 [35.0, 463]	93.0 [7.00, 528]
Total sum (%)	2427 (16.9%)	5672 (39.6%)	1152 (8.1%)	5064 (35.4%)	14315 (100%)
**Seedbank change**					
Mean (SD)	-0.425 (0.534)	0.337 (0.358)	0.264 (0.630)	-0.0906 (0.638)	0.0885 (0.609)
Median [Min, Max]	-0.293 [-1.18, 0.722]	0.404 [-0.318, 0.990]	0.373 [-1.04, 1.22]	-0.0313 [-1.10, 1.38]	0.167 [-1.18, 1.38]

Supp. Mat. Figure S3. Initial against follow-up seedbank, with associated boxplot for the 59 fields with colours indicating the country. The dashed straight line corresponds to the situation where initial = follow-up seedbank. The points below this line correspond to a reduction of the seedbank size, and the points above correspond to an increase of the seedbank size. The plot is created using R version 3.6.1[1] and the package 'ggpubr'[11].

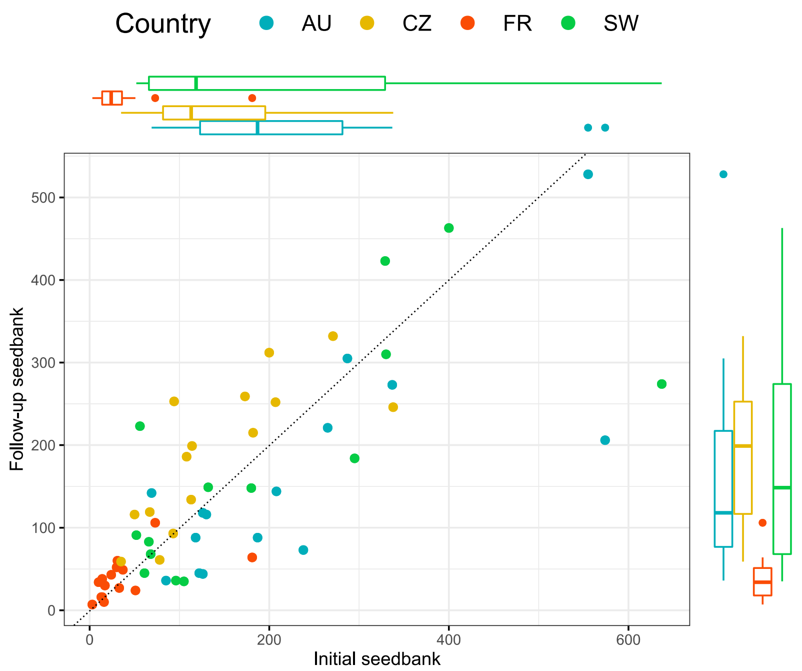

Seed card description:

Supp. Mat. Table S4. Descriptive statistics (mean (SD) and median [min, max]) for the average number of seeds eaten and seed predation rate per field and session. Sixteen seed cards were exposed per field, with fifty *Poa annua* L. seeds on each card, leading to 800 seeds exposed per field and session during 7 days. All countries included 15 fields with two sampling sessions (n = 30).

	AU (n=30)	CZ (n=30)	FR (n=30)	SW (n=30)	Overall (n=120)
**Number of seeds eaten / field and session**					
Mean (SD)	149 (98.2)	190 (201)	76.0 (102)	98.4 (76.5)	128 (135)
Median	121	90.0	48.5	75.0	75.5
[Min, Max]	[36.0, 477]	[7.00, 721]	[7.00, 480]	[15.0, 328]	[7.00, 721]
**Predation rate / field and session**					
Mean (SD)	0.187 (0.123)	0.238 (0.252)	0.0951 (0.127)	0.125 (0.0952)	0.161 (0.169)
Median	0.151	0.113	0.0606	0.0994	0.0956
[Min, Max]	[0.0450, 0.596]	[0.00875, 0.901]	[0.00933, 0.600]	[0.0188, 0.410]	[0.00875, 0.901]

Supp. Mat. Figure S4. Weed seed predation rate (seed cards) according to (a) the countries and (b) the sampling session. Letters indicates significant differences between sessions, and are based on the model with granivores explaining seed predation rates presented in Supp. Mat. Table S11. The plot is created using R version 3.6.1[1] and the package 'ggplot2'[2].

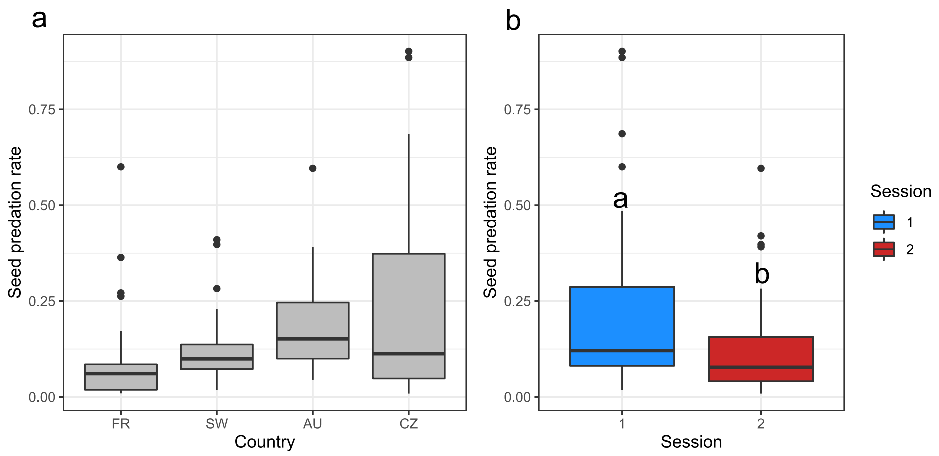

Supp. Mat. Table S5. Descriptive statistics (mean (SD) and median [min, max]) for the per capita seed consumption (PCSC) per field and session for the different carabid groups. The PCSC was calculated as the number of seeds consumed divided by the number of carabids captured per field and session (for each carabid group separately).

	**AU (n=30)**	**CZ (n=30)**	**FR (n=30)**	**SW (n=30)**	**Overall (n=120)**
**PCSC all carabids**					
Mean (SD)	0.228 (0.300)	0.493 (0.423)	0.222 (0.223)	0.274 (0.340)	0.304 (0.344)
Median [Min, Max]	0.140 [0.050, 1.60]	0.349 [0.019, 1.30]	0.163 [0.018, 0.919]	0.142 [0.032, 1.50]	0.173 [0.018, 1.60]
**PCSC seed-eating carabids**					
Mean (SD)	0.327 (0.384)	0.931 (0.932)	0.620 (0.626)	0.328 (0.402)	0.551 (0.668)
Median [Min, Max]	0.187 [0.055, 2.05]	0.521 [0.055, 3.34]	0.503 [0.038, 2.81]	0.183 [0.038, 1.70]	0.261 [0.038, 3.34]
**PCSC granivores**					
Mean (SD)	7.63 (9.68)	11.7 (14.6)	8.88 (8.34)	3.30 (3.78)	7.88 (10.2)
Median [Min, Max]	4.01 [0.596, 38.7]	5.24 [0.400, 51.6]	5.71 [0.737, 32.7]	1.86 [0.229, 14.7]	4.03 [0.229, 51.6]
**PCSC omnivores**					
Mean (SD)	1.08 (4.16)	1.18 (1.23)	0.727 (0.818)	0.522 (0.811)	0.879 (2.23)
Median [Min, Max]	0.221 [0.061, 23.0]	0.622 [0.056, 4.37]	0.555 [0.040, 3.64]	0.202 [0.039, 3.95]	0.295 [0.039, 23.0]

Description of alternative prey :

Supp. Mat. Table S6. Correspondence between the index used to estimate the number of Collembola and the range of abundance per sample. We then converted this index into abundance by taking the mean of the corresponding range, then into biomass.

Collembola range	Index
0	1
1	2
2	3
3 to 4	4
5 to 8	5
9 to 16	6
17 to 32	7
33 to 64	8
65 to 128	9
129 to 256	10

257 to 512	11
513 to 1024	12
1025 to 2048	13

Supp. Mat. Table S7. Description of the alternative prey sampled in experiment (two sessions for all countries with the exception of Austria). We detailed the field occurrence (number of fields with presence and the corresponding percentage), the abundance (mean (SD), median [min, max] and the total abundance) and the biomass (mean (SD), median [min, max] and the total biomass) for all Arachnida, Aphididae, Collembola and the sum of these three groups. We converted the counts into biomass using equations that derive mass from body length[12,13] for European Arachnida[14], and using estimation of individual masses from the literature for Collembola[15] and Aphididae[16].

	**AU (n=15)**	**CZ (n=30)**	**FR (n=30)**	**SW (n=30)**	**Overall (n=105)**
**Arachnida**					
**Occurrence**	15 (100%)	30 (100%)	30 (100%)	30 (100%)	105 (100%)
**Abundance**					
Mean (SD)	13.6 (15.3)	70.1 (97.7)	32.8 (23.0)	28.5 (17.0)	39.5 (57.8)
Median [Min, Max]	7.00 [2.00, 62.0]	36.0 [10.0, 437]	27.5 [4.00, 94.0]	25.0 [1.00, 82.0]	26.0 [1.00, 437]
Sum	205	2104	984	877	4170
**Biomass (mg)**					
Mean (SD)	7.77 (5.35)	94.5 (161)	26.5 (19.8)	18.2 (10.8)	40.9 (92.7)
Median [Min, Max]	5.38 [3.06, 19.3]	37.7 [8.80, 715]	19.9 [4.46, 89.2]	15.8 [0.682, 48.3]	18.6 [0.682, 715]
Sum	116.62	2835.80	796.23	546.88	4295.55
**Aphididae**					
**Occurrence**	15 (100%)	30 (100%)	30 (100%)	30 (100%)	105 (100%)
**Abundance**					
Mean (SD)	1100 (1850)	135 (128)	66.6 (69.9)	628 (879)	394 (906)
Median [Min, Max]	209 [32.0, 6670]	89.0 [28.0, 646]	43.0 [14.0, 349]	314 [53.0, 3950]	107 [14.0, 6670]
Sum	16459	4049	1999	18840	41347
**Biomass (mg)**					
Mean (SD)	4940 (8330)	607 (578)	300 (314)	2830 (3950)	1770 (4080)
Median [Min, Max]	941 [144, 30000]	401 [126, 2910]	194 [63.0, 1570]	1410 [239, 17800]	482 [63.0, 30000]
Sum	74065.5	17982	8995.5	81909	182952
**Collembola**					
**Occurrence**	15 (100%)	30 (100%)	30 (100%)	26 (86.6%)	101 (96.1%)
**Abundance**					
Mean (SD)	4350 (3500)	1450 (1020)	4720 (2010)	52.7 (129)	2400 (2650)
Median [Min, Max]	3370 [632, 11100]	1160 [494, 5580]	4660 [1350, 8650]	6.50 [0.00, 542]	1330 [0.00, 11100]
Sum	65237	43487	141494	1582	251800
**Biomass (mg)**					
Mean (SD)	358 (288)	119 (84.2)	388 (166)	4.34 (10.6)	198 (218)
Median [Min, Max]	277 [52.1, 918]	95.2 [40.7, 459]	384 [111, 712]	0.535 [0.00, 44.6]	109 [0.00, 918]
Sum	5372.9	3426.7	11653.5	129.9	20583.2
**Sum of the three groups**					
**Abundance**					
Mean (SD)	5460 (3880)	1650 (1050)	4820 (2000)	709 (898)	2830 (2740)
Median [Min, Max]	4570 [777, 13200]	1350 [634, 5700]	4790 [1400, 8860]	369 [65.0, 4040]	1830 [65.0, 13200]
Sum	81900	49640	144477	21276	297293
**Biomass (mg)**					
Mean (SD)	5300 (8330)	821 (587)	715 (350)	2850 (3950)	2010 (4060)
Median [Min, Max]	1140 [425, 30100]	668 [208, 3110]	644 [238, 1770]	1460 [250, 17800]	813 [208, 30100]
Sum	79555.06	24637.91	21445.22	85457.18	211095.4

Supp. Mat. Table S8. Description of the Arachnida trapped in experiment (two sessions for all countries with the exception of Austria). We detailed the total captured (number of individuals), the number of individual captured per field (mean (SD); median [min, max]) and the field occurrence (number of fields with presence and the corresponding percentage).

Group of prey	Austria(n = 15)				The Czech Republic (n = 30)				France (n = 30)				Sweden (n = 30)				Overall (n = 105)			
	Total	Mean (SD)	Median [Min; Max]	Occurrence	Total	Mean (SD)	Median [Min; Max]	Occurrence	Total	Mean (SD)	Median [Min; Max]	Occurrence	Total	Mean (SD)	Median [Min; Max]	Occurrence	Total	Mean (SD)	Median [Min; Max]	Occurrence
Agelenidae	1	0.07 (0.26)	0.00 [0.00, 1.00]	1 (6.6%)	1	0.03 (0.18)	0.00 [0.00, 1.00]	1 (3.3%)									2	0.02 (0.14)	0.00 [0.00, 1.00]	2 (1.9%)
Araneidae	5	0.33 (0.62)	0.00 [0.00, 2.00]	4 (26.6%)	987	32.9 (79.1)	4.00 [0.00, 356]	25 (83.3%)	124	4.13 (4.86)	2.00 [0.00, 21.0]	25 (83.3%)	5	0.17 (0.46)	0.00 [0.00, 2.00]	4 (13.3%)	1121	10.7 (44.2)	1.00 [0.00, 356]	58 (55.2%)
Clubionidae					8	0.27 (0.58)	0.00 [0.00, 2.00]	6 (20%)	2	0.07 (0.25)	0.00 [0.00, 1.00]	2 (6.6%)					10	0.1 (0.35)	0.00 [0.00, 2.00]	8 (7.6%)
Dictynidae					1	0.03 (0.18)	0.00 [0.00, 1.00]	1 (3.3%)	1	0.03 (0.18)	0.00 [0.00, 1.00]	1 (3.3%)					2	0.02 (0.14)	0.00 [0.00, 1.00]	2 (1.9%)
Gnaphosidae					7	0.23 (0.57)	0.00 [0.00, 2.00]	5 (16.6%)					1	0.03 (0.18)	0.00 [0.00, 1.00]	1 (3.3%)	8	0.08 (0.33)	0.00 [0.00, 2.00]	6 (5.7%)
Linyphiidae	112	7.47 (6.89)	6.00 [0.00, 25.0]	13 (86.6%)	425	14.2 (10.7)	13.0 [1.00, 45.0]	30 (100%)	566	18.9 (13.8)	15.5 [2.00, 47.0]	30 (100%)	658	21.9 (14.2)	19.5 [0.00, 67.0]	29 (96.6%)	1761	16.8 (13.1)	15.0 [0.00, 67.0]	102 (97.1%)
Lycosidae	6	0.4 (0.83)	0.00 [0.00, 3.00]	4 (26.6%)	152	5.07 (9.48)	2.00 [0.00, 45.0]	19 (63.3%)	91	3.03 (6.7)	0.00 [0.00, 28.0]	13 (43.3%)	64	2.13 (2)	2.00 [0.00, 6.00]	22 (73.3%)	313	2.98 (6.42)	1.00 [0.00, 45.0]	58 (55.2%)
Mimetidae					1	0.03 (0.18)	0.00 [0.00, 1.00]	1 (3.3%)									1	0.01 (0.1)	0.00 [0.00, 1.00]	1 (0.9%)
Miturgidae					1	0.03 (0.18)	0.00 [0.00, 1.00]	1 (3.3%)					1	0.03 (0.18)	0.00 [0.00, 1.00]	1 (3.3%)	2	0.02 (0.14)	0.00 [0.00, 1.00]	2 (1.9%)
Opiliones					5	0.17 (0.46)	0.00 [0.00, 2.00]	4 (13.3%)	5	0.17 (0.46)	0.00 [0.00, 2.00]	4 (13.3%)	2	0.07 (0.25)	0.00 [0.00, 1.00]	2 (6.6%)	12	0.11 (0.38)	0.00 [0.00, 2.00]	10 (9.5%)
Philodromidae					16	0.53 (1.25)	0.00 [0.00, 5.00]	7 (23.3%)	1	0.03 (0.18)	0.00 [0.00, 1.00]	1 (3.3%)					17	0.16 (0.71)	0.00 [0.00, 5.00]	8 (7.6%)
Phrurolithidae					2	0.07 (0.25)	0.00 [0.00, 1.00]	2 (6.6%)									2	0.02 (0.14)	0.00 [0.00, 1.00]	2 (1.9%)
Pisauridae					52	1.73 (2.43)	0.500 [0.00, 9.00]	15 (50%)	2	0.07 (0.25)	0.00 [0.00, 1.00]	2 (6.6%)					54	0.51 (1.51)	0.00 [0.00, 9.00]	17 (16.1%)
Pseudoscorpiones	55	3.67 (14.2)	0.00 [0.00, 55.0]	1 (6.6%)	1	0.03 (0.18)	0.00 [0.00, 1.00]	1 (3.3%)	61	2.03 (3.12)	0.00 [0.00, 10.0]	14 (46.6%)					117	1.11 (5.64)	0.00 [0.00, 55.0]	16 (15.2%)
Salticidae					6	0.2 (0.61)	0.00 [0.00, 3.00]	4 (13.3%)	3	0.1 (0.4)	0.00 [0.00, 2.00]	2 (6.6%)	3	0.1 (0.4)	0.00 [0.00, 2.00]	2 (6.6%)	12	0.11 (0.45)	0.00 [0.00, 3.00]	8 (7.6%)
Tetragnahidae	20	1.33 (1.35)	1.00 [0.00, 4.00]	10 (66.6%)	65	2.17 (1.88)	2.00 [0.00, 6.00]	25 (83.3%)	13	0.43 (0.68)	0.00 [0.00, 2.00]	10 (33.3%)	9	0.3 (0.84)	0.00 [0.00, 4.00]	5 (16.6%)	107	1.02 (1.48)	0.00 [0.00, 6.00]	50 (47.6%)
Theridiidae	5	0.33 (0.49)	0.00 [0.00, 1.00]	5 (33.3%)	239	7.97 (7.16)	6.00 [1.00, 28.0]	30 (100%)	47	1.57 (1.28)	2.00 [0.00, 4.00]	21 (70%)	47	1.57 (1.91)	1.00 [0.00, 8.00]	19 (63.3%)	338	3.22 (5.01)	1.00 [0.00, 28.0]	75 (71.4%)
Thomisidae					135	4.5 (7.48)	1.00 [0.00, 35.0]	18 (60%)	68	2.27 (2.74)	1.50 [0.00, 9.00]	19 (63.3%)	40	1.33 (3.16)	0.00 [0.00, 17.0]	14 (46.6%)	243	2.31 (4.79)	0.00 [0.00, 35.0]	51 (48.5%)
Unidentified													24	0.8 (1.73)	0.00 [0.00, 8.00]	10 (33.3%)	24	0.23 (0.98)	0.00 [0.00, 8.00]	10 (9.5%)

Supp. Mat. Table S9. Description of all alternative prey trapped in experiment (two sessions for all countries with the exception of Austria). We detailed the mean (standard deviation) and the total captured (sum of the number of individuals) for each group per country.

	**Austria (n=15)**		**Czech Republic (n=30)**		**France (n=30)**		**Sweden (n=30)**		**Overall (n=105)**	
	**Mean (SD)**	**Sum**	**Mean (SD)**	**Sum**	**Mean (SD)**	**Sum**	**Mean (SD)**	**Sum**	**Mean (SD)**	**Sum**
**Collembola**	4350 (3500)	141494	1450 (1020)	65237	4720 (2010)	43487	52.7 (129)	1582	2400 (2650)	251800 (71.69%)
**Aphididae**	1100 (1850)	1999	135 (128)	16459	66.6 (69.9)	4049	628 (879)	18840	394 (906)	41347 (11.77%)
Thysanoptera	72.0 (36.2)	7310	121 (84.2)	1080	244 (162)	3644	155 (108)	4651	159 (128)	16685 (4.75%)
Hymenoptera (except Formicidae and symphita larvae)	68.1 (35.9)	1881	72.2 (51.5)	1022	62.7 (52.4)	2166	153 (88.4)	4580	91.9 (73.2)	9649 (2.75%)
Nematocera	62.1 (35.9)	4813	52.8 (59.9)	932	160 (155)	1583	2.03 (3.62)	61	70.4 (108)	7389 (2.1%)
Auchenorrhyncha	8.20 (6.68)	552	108 (95.4)	123	18.4 (21.7)	3250	24.7 (29.2)	740	44.4 (67.7)	4665 (1.33%)
Brachycera	27.1 (12.4)	973	60.4 (73.9)	407	32.4 (29.0)	1812	33.5 (48.7)	1005	40.0 (51.1)	4197 (1.19%)
**Arachnida**	13.6 (15.3)	984	70.1 (97.7)	204	32.8 (23.0)	2104	28.5 (17.0)	854	39.5 (57.8)	4146 (1.18%)
Chrysomelidae	39.9 (45.0)	766	21.2 (25.7)	598	25.5 (32.4)	635	16.4 (28.1)	493	23.7 (32.0)	2492 (0.71%)
Other coleoptera	0.467 (0.834)	174	41.0 (23.9)	7	5.80 (6.89)	1229	9.50 (8.14)	285	16.1 (21.2)	1695 (0.48%)
Staphylinidae	1.73 (1.98)	384	22.9 (19.1)	26	12.8 (11.8)	686	19.0 (25.0)	571	15.9 (19.1)	1667 (0.47%)
Other larvae	33.7 (17.2)	826	2.47 (5.46)	506	27.5 (32.2)	74	4.57 (5.75)	137	14.7 (22.8)	1543 (0.4%)
Heteroptera	18.6 (19.7)	40	16.9 (11.6)	279	1.33 (1.81)	506	17.6 (11.7)	529	12.9 (13.5)	1354 (0.39%)
Coccinellidae	0.00 (0.00)	4	5.23 (9.65)	0	0.133 (0.434)	157	21.4 (22.2)	643	7.66 (15.6)	804 (0.23%)
Egg, cocoon, pupae	2.40 (4.52)	459	0.400 (1.61)	36	15.3 (27.8)	12	0.300 (0.952)	9	4.91 (16.2)	516 (0.15%)
Formicidae	3.87 (6.40)	74	4.13 (6.82)	58	2.47 (3.29)	124	3.73 (7.75)	112	3.50 (6.22)	368 (0.1%)
Neuroptera	0.00 (0.00)	1	1.53 (2.06)	0	0.0333 (0.183)	46	6.80 (5.30)	204	2.39 (4.16)	251 (0.07%)
Lepidoptera	1.07 (1.44)	19	1.03 (1.30)	16	0.633 (1.10)	31	0.733 (1.31)	22	0.838 (1.26)	88 (0.03%)
Curculionidae	0.933 (1.49)	6	0.367 (0.490)	14	0.200 (0.551)	11	0.967 (1.54)	29	0.571 (1.11)	60 (0.02%)
Gastropoda	0.00 (0.00)	41	0.467 (0.776)	0	1.37 (2.39)	14	0.133 (0.346)	4	0.562 (1.44)	59 (0.02%)
Diplura	0.00 (0.00)	57	0.00 (0.00)	0	1.90 (4.56)	0	0.00 (0.00)	0	0.543 (2.56)	57 (0.02%)
Unidentifiable prey	1.47 (1.55)	4	0.767 (4.20)	22	0.133 (0.434)	23	0.0333 (0.183)	1	0.476 (2.36)	50 (0.01%)
Cantharidae	0.400 (0.828)	11	0.800 (1.52)	6	0.367 (0.809)	24	0.267 (0.521)	8	0.467 (1.02)	49 (0.01%)
Chilopoda	0.200 (0.561)	6	1.00 (1.80)	3	0.200 (0.407)	30	0.233 (0.679)	7	0.438 (1.12)	46 (0.01%)
Symphyta larvae	0.800 (0.775)	26	0.100 (0.305)	12	0.867 (1.57)	3	0.00 (0.00)	0	0.390 (0.976)	41 (0.01%)
Isopoda	0.667 (2.58)	7	0.367 (0.718)	10	0.233 (0.504)	11	0.300 (0.837)	9	0.352 (1.15)	37 (0.01%)
Diplopoda	0.267 (0.799)	6	0.500 (0.861)	4	0.200 (0.664)	15	0.333 (1.06)	10	0.333 (0.862)	35 (0.01%)
Dermaptera	0.533 (1.81)	7	0.467 (1.85)	8	0.233 (0.568)	14	0.00 (0.00)	0	0.276 (1.24)	29 (0.01%)
Elateridae	0.400 (0.828)	7	0.200 (0.610)	6	0.233 (0.568)	6	0.333 (1.12)	10	0.276 (0.803)	29 (0.01%)
Psocoptera	0.00 (0.00)	9	0.600 (1.19)	0	0.300 (0.596)	18	0.00 (0.00)	0	0.257 (0.747)	27 (0.01%)
Sternorrhyncha	0.933 (1.98)	1	0.100 (0.305)	14	0.0333 (0.183)	3	0.00 (0.00)	0	0.171 (0.814)	18 (0.01%)
Other	0.00 (0.00)	0	0.00 (0.00)	0	0.00 (0.00)	0	0.333 (1.65)	10	0.0952 (0.883)	10 (0%)
Silphidae	0.00 (0.00)	0	0.00 (0.00)	0	0.00 (0.00)	0	0.233 (0.626)	7	0.0667 (0.347)	7 (0%)
Orthoptera	0.00 (0.00)	3	0.0667 (0.254)	0	0.100 (0.305)	2	0.00 (0.00)	0	0.0476 (0.214)	5 (0%)
Blattidae	0.00 (0.00)	2	0.00 (0.00)	0	0.0667 (0.365)	0	0.00 (0.00)	0	0.0190 (0.195)	2 (0%)
Enchytraeidae	0.00 (0.00)	2	0.00 (0.00)	0	0.0667 (0.365)	0	0.00 (0.00)	0	0.0190 (0.195)	2 (0%)
Nematoda	0.00 (0.00)	1	0.00 (0.00)	0	0.0333 (0.183)	0	0.00 (0.00)	0	0.00952 (0.0976)	1 (0%)

Effect of the different groups of carabids on the weed seedbank change and modulation by the alternative prey

Supp. Mat. Table S10. Results of linear models (LMs) relating the follow-up seedbank to the initial seedbank, proportion of arable crops, pesticide intensity and AD of carabids alone or in interaction with alternative prey biomass. Five different analysis were conducted to test the effect of carabids (Std. Error) alone on seedbank change and to assess the interaction effect of carabids (Std. Error) with alternative prey (Aphididae, Collembola, Arachnida and the total of alternative prey). We fitted models for sessions 1 and 2 separately. For each model we reported the corresponding session with the number of observation (n), the AIC, the R-squared (R^2), and for each variable in the model we reported the estimate with standard error (Est. (Std. Error)), the F-value (F), the degree of freedom (Df) and the P-value. In addition, we added the analysis of the contribution of carnivores to the regulation of the seed bank.

Analyses	Session	AIC	R^2	Explanatory variable	Est. (Std. Error)	F	Df	P-value)
**All carabids**								
Effect of all carabids	1 (n = 57)	103.3	0.68	log(All carabids)	0.01 (0.11)	0.00	1	0.952
				log(Initial seedbank)	0.71 (0.08)	73.43	1	<0.001***
				PesticideIntensity	-0.03 (0.05)	0.48	1	0.492
				pCrop	0.54 (0.43)	1.58	1	0.214
	2 (n = 57)	92.4	0.73	log(All carabids)	-0.31 (0.9)	10.89	1	0.002**
				log(Initial seedbank)	0.73 (0.07)	98.18	1	<0.001***
				PesticideIntensity	-0.04 (0.04)	1.02	1	0.317
				pCrop	0.13 (0.41)	0.10	1	0.756
Effect of all carabids x aphids	1 (n = 57)	98.7	0.72	log(All carabids) : log(Aphid)	0.04 (0.11)	0.16	1	0.694
				log(All carabids)	-0.24 (0.68)	0.05	1	0.817
				log(Aphid)	-0.43 (0.68)	7.93	1	0.007**
				log(Initial seedbank)	0.75 (0.08)	84.54	1	<0.001***
				PesticideIntensity	-0.08 (0.05)	2.70	1	0.107
				pCrop	0.72 (0.41)	3.01	1	0.089.
	2 (n = 42)	72.6	0.76	log(All carabids) : log(Aphid)	-0.01 (0.19)	0.00	1	0.973
				log(All carabids)	-0.18 (1.14)	2.71	1	0.109
				log(Aphid)	0.03 (1.08)	0.01	1	0.939
				log(Initial seedbank)	0.72 (0.09)	65.98	1	<0.001***
				PesticideIntensity	-0.09 (0.06)	2.17	1	0.150
				pCrop	-0.29 (0.51)	0.31	1	0.581
Effect of all carabids x collembola	1 (n = 57)	106.8	0.67	log(All carabids) : log(Collembola+0.5)	0.05 (0.07)	0.44	1	0.511
				log(All carabids)	-0.24 (0.41)	0.01	1	0.909
				log(Collembola+0.5)	-0.32 (0.50)	0.02	1	0.896
				log(Initial seedbank)	0.70 (0.09)	64.86	1	<0.001***
				PesticideIntensity	-0.03 (0.05)	0.43	1	0.514
				pCrop	0.56 (0.51)	1.23	1	0.273
	2 (n = 42)	65.8	0.79	log(All carabids) : log(Collembola+0.5)	0.01 (0.07)	0.05	1	0.832
				log(All carabids)	-0.27 (0.32)	2.71	1	0.109
				log(Collembola+0.5)	0.01 (0.41)	6.15	1	0.018*
				log(Initial seedbank)	0.77 (0.08)	100.88	1	<0.001***
				PesticideIntensity	-0.14 (0.06)	6.12	1	0.018*
				pCrop	0 (0.48)	0.00	1	0.999
Effect of all carabids x arachnida	1 (n = 57)	102.4	0.7	log(All carabids) : log(Arachnida)	-0.30 (0.18)	3.01	1	0.089.
				log(All carabids)	0.88 (0.51)	0.03	1	0.868
				log(Arachnida)	2.08 (1.12)	1.41	1	0.240
				log(Initial seedbank)	0.68 (0.09)	61.79	1	<0.001***
				PesticideIntensity	-0.05 (0.05)	0.99	1	0.324
				pCrop	0.52 (0.43)	1.51	1	0.225
	2 (n = 42)	70.5	0.77	log(All carabids) : log(Arachnida)	0.11 (0.12)	0.87	1	0.358
				log(All carabids)	-0.66 (0.50)	2.58	1	0.117
				log(Arachnida)	-0.53 (0.65)	0.94	1	0.338
				log(Initial seedbank)	0.70 (0.08)	79.23	1	<0.001***
				PesticideIntensity	-0.09 (0.06)	2.49	1	0.123
				pCrop	-0.07 (0.54)	0.02	1	0.897
		96.9	0.72	log(All carabids) : log(Total prey)	0.20 (0.16)	1.66	1	0.203

Effect of all carabids x total prey	1 (n = 57)			log(All carabids)	-1.40 (1.08)	0.02	1	0.896
				log(Total prey)	-1.48 (1.0)	8.37	1	0.006**
				log(Initial seedbank)	0.72 (0.08)	85.05	1	<0.001***
				PesticideIntensity	-0.08 (0.05)	2.81	1	0.100.
				pCrop	0.59 (0.40)	2.21	1	0.143
	2 (n = 42)	69	0.78	log(All carabids) : log(Total prey)	0.34 (0.28)	1.41	1	0.243
				log(All carabids)	-2.11 (1.86)	2.65	1	0.113
				log(Total prey)	-1.73 (1.62)	1.71	1	0.199
				log(Initial seedbank)	0.67 (0.08)	67.42	1	<0.001***
				PesticideIntensity	-0.11 (0.06)	3.84	1	0.058.
				pCrop	-0.05 (0.49)	0.01	1	0.918
Granivore carabids								
Effect of granivores	1 (n = 57)	101.7	0.69	log(Granivore+0.5)	0.09 (0.08)	1.46	1	0.230
				log(Initial seedbank)	0.68 (0.08)	65.91	1	<0.001***
				PesticideIntensity	-0.02 (0.05)	0.12	1	0.733
				pCrop	0.35 (0.45)	0.6	1	0.442
	2 (n = 57)	102	0.69	log(Granivore+0.5)	-0.07 (0.06)	1.17	1	0.285
				log(Initial seedbank)	0.74 (0.08)	79.73	1	<0.001***
				PesticideIntensity	-0.06 (0.05)	1.13	1	0.293
				pCrop	0.64 (0.43)	2.19	1	0.145
Effect of granivores x aphids	1 (n = 57)	97.2	0.72	log(Granivore+0.5) : log(Aphid)	-0.02 (0.05)	0.12	1	0.727
				log(Granivore)	0.20 (0.35)	1.42	1	0.239
				log(Aphid)	-0.10 (0.16)	7.84	1	0.007**
				log(Initial seedbank)	0.71 (0.08)	75.99	1	<0.001***
				PesticideIntensity	-0.06 (0.05)	1.48	1	0.229
				pCrop	0.54 (0.43)	1.52	1	0.223
	2 (n = 42)	73.1	0.76	log(Granivore+0.5) : log(Aphid)	0.05 (0.10)	0.32	1	0.576
				log(Granivore+0.5)	-0.44 (0.59)	2.01	1	0.165
				log(Aphid)	-0.12 (0.23)	0.00	1	0.959
				log(Initial seedbank)	0.75 (0.09)	65.69	1	<0.001***
				PesticideIntensity	-0.14 (0.06)	5.87	1	0.021*
				pCrop	-0.13 (0.51)	0.07	1	0.798
Effect of granivores x collembola	1 (n = 57)	104.9	0.68	log(Granivore+0.5) : log(Collembola+0.5)	-0.02 (0.03)	0.58	1	0.449
				log(Granivore)	0.20 (0.16)	1.52	1	0.223
				log(Collembola+0.5)	0.09 (0.12)	0.07	1	0.790
				log(Initial seedbank)	0.71 (0.09)	59.92	1	<0.001***
				PesticideIntensity	-0.02 (0.05)	0.09	1	0.772
				pCrop	0.29 (0.51)	0.33	1	0.568
	2 (n = 42)	66.3	0.79	log(Granivore+0.5) : log(Collembola+0.5)	-0.05 (0.04)	1.72	1	0.198
				log(Granivore)	0.18 (0.19)	0.60	1	0.446
				log(Collembola+0.5)	0.26 (0.14)	4.78	1	0.036*
				log(Initial seedbank)	0.82 (0.09)	89.60	1	<0.001***
				PesticideIntensity	-0.16 (0.06)	7.58	1	0.009**
				pCrop	-0.24 (0.55)	0.19	1	0.666
Effect of granivores x arachnida	1 (n = 57)	103.7	0.69	log(Granivore+0.5) : log(Arachnida)	-0.01 (0.11)	0.01	1	0.933
				log(Granivore)	0.13 (0.32)	1.87	1	0.178
				log(Arachnida)	0.19 (0.33)	1.74	1	0.194
				log(Initial seedbank)	0.64 (0.09)	52.21	1	<0.001***
				PesticideIntensity	-0.04 (0.05)	0.53	1	0.470
				pCrop	0.22 (0.47)	0.22	1	0.642
	2 (n = 42)	72.8	0.76	log(Granivore+0.5) : log(Arachnida)	-0.01 (0.10)	0.00	1	0.960
				log(Granivore)	-0.07 (0.39)	1.43	1	0.241
				log(Arachnida)	0.07 (0.29)	0.55	1	0.465
				log(Initial seedbank)	0.73 (0.09)	70.97	1	<0.001***
				PesticideIntensity	-0.13 (0.07)	3.79	1	0.060.
				pCrop	0.04 (0.61)	0.01	1	0.943
Effect of granivores x total prey	1 (n = 57)	97.3	0.72	log(Granivore+0.5) : log(Total prey)	-0.01 (0.06)	0.05	1	0.828
				log(Granivore)	0.17 (0.43)	1.19	1	0.281
				log(Total prey)	-0.15 (0.20)	7.81	1	0.007**
				log(Initial seedbank)	0.68 (0.08)	73.26	1	<0.001***
				PesticideIntensity	-0.05 (0.05)	1.08	1	0.304
				pCrop	0.42 (0.43)	0.96	1	0.332
	2 (n = 42)	71.7	0.76	log(Granivore+0.5) : log(Total prey)	0.04 (0.13)	0.10	1	0.752
				log(Granivore)	-0.36 (0.83)	1.53	1	0.225
				log(Total prey)	0.08 (0.34)	1.34	1	0.255
				log(Initial seedbank)	0.71 (0.09)	61.25	1	<0.001***
				PesticideIntensity	-0.14 (0.06)	5.94	1	0.020*
				pCrop	0.02 (0.51)	0.00	1	0.971

Omnivore carabids								
Effect of omnivores	1 (n = 57)	103.3	0.68	log(Omnivore)	-0.01 (0.08)	0.02	1	0.882
				log(Initial seedbank)	0.72 (0.08)	75.28	1	<0.001***
				PesticideIntensity	-0.04 (0.05)	0.54	1	0.467
				pCrop	0.53 (0.42)	1.57	1	0.215
	2 (n = 57)	95.8	0.72	log(Omnivore)	-0.23 (0.09)	7.31	1	0.009**
				log(Initial seedbank)	0.75 (0.08)	95.41	1	<0.001***
				PesticideIntensity	-0.05 (0.05)	1.03	1	0.316
				pCrop	0.24 (0.41)	0.35	1	0.556
Effect of omnivores x aphids	1 (n = 57)	98.9	0.71	log(Omnivore) : log(Aphid)	0.0 (0.07)	0.00	1	0.965
				log(Omnivore)	0.0 (0.46)	0.10	1	0.758
				log(Aphid)	-0.18 (0.42)	7.94	1	0.007**
				log(Initial seedbank)	0.74 (0.08)	78.60	1	<0.001***
				PesticideIntensity	-0.8 (0.05)	2.28	1	0.137
				pCrop	0.69 (0.41)	2.80	1	0.100
	2 (n = 42)	73.7	0.75	log(Omnivore) : log(Aphid)	0.01 (0.15)	0.01	1	0.939
				log(Omnivore)	-0.21 (0.91)	1.71	1	0.199
				log(Aphid)	-0.05 (0.78)	0.01	1	0.926
				log(Initial seedbank)	0.73 (0.09)	65.83	1	<0.001***
				PesticideIntensity	-0.10 (0.06)	2.89	1	0.098.
				pCrop	-0.27 (0.52)	0.27	1	0.609
Effect of omnivores x collembola	1 (n = 57)	105.5	0.68	log(Omnivore) : log(Collembola+0.5)	0.06 (0.05)	1.60	1	0.212
				log(Omnivore)	-0.33 (0.27)	0.01	1	0.905
				log(Collembola+0.5)	-0.36 (0.29)	0.00	1	0.996
				log(Initial seedbank)	0.70 (0.08)	67.65	1	<0.001***
				PesticideIntensity	-0.04 (0.05)	0.45	1	0.504
				pCrop	0.50 (0.50)	1.01	1	0.319
	2 (n = 42)	67.2	0.79	log(Omnivore) : log(Collembola+0.5)	0.0 (0.06)	0.00	1	0.949
				log(Omnivore)	-0.14 (0.24)	1.51	1	0.228
				log(Collembola+0.5)	0.07 (0.31)	5.92	1	0.020*
				log(Initial seedbank)	0.78 (0.08)	96.01	1	<0.001***
				PesticideIntensity	-0.16 (0.06)	7.19	1	0.011*
				pCrop	0.02 (0.51)	0.00	1	0.975
Effect of omnivores x arachnida	1 (n = 57)	102.8	0.69	log(Omnivore) : log(Arachnida)	-0.21 (0.13)	2.67	1	0.109
				log(Omnivore)	0.58 (0.37)	0.02	1	0.894
				log(Arachnida)	1.33 (0.73)	1.37	1	0.247
				log(Initial seedbank)	0.69 (0.09)	65.73	1	<0.001***
				PesticideIntensity	-0.05 (0.05)	0.68	1	0.413
				pCrop	0.51 (0.42)	1.46	1	0.232
	2 (n = 42)	71.8	0.76	log(Omnivore) : log(Arachnida)	0.08 (0.10)	0.62	1	0.436
				log(Omnivore)	-0.46 (0.42)	1.65	1	0.208
				log(Arachnida)	-0.30 (0.49)	1.03	1	0.317
				log(Initial seedbank)	0.73 (0.08)	75.64	1	<0.001***
				PesticideIntensity	-0.11 (0.05)	3.69	1	0.063.
				pCrop	-0.07 (0.55)	0.02	1	0.895
Effect of omnivores x total prey	1 (n = 57)	96.9	0.72	log(Omnivore) : log(Total prey)	0.14 (0.11)	1.65	1	0.205
				log(Omnivore)	-0.99 (0.77)	0.01	1	0.915
				log(Total prey)	-1.01 (0.64)	8.34	1	0.006**
				log(Initial seedbank)	0.73 (0.08)	87.01	1	<0.001***
				PesticideIntensity	-0.08 (0.05)	2.82	1	0.099.
				pCrop	0.61 (0.40)	2.33	1	0.133
	2 (n = 42)	70.4	0.77	log(Omnivore) : log(Total prey)	0.16 (0.21)	0.56	1	0.457
				log(Omnivore)	-1.20 (1.39)	2.22	1	0.145
				log(Total prey)	-0.60 (1.11)	2.32	1	0.137
				log(Initial seedbank)	0.69 (0.08)	67.31	1	<0.001***
				PesticideIntensity	-0.12 (0.06)	4.10	1	0.051.
				pCrop	-0.10 (0.50)	0.04	1	0.846
Carnivore carabids								
Effect of carnivores	1 (n = 59)			log(Carnivore)	0.06 (0.10)	0.33	1	0.569
				log(Initial seedbank)	0.71 (0.08)	76.94	1	<0.001***
				PesticideIntensity	-0.04 (0.05)	0.63	1	0.432
				pCrop	0.61 (0.44)	1.90	1	0.174
	2 (n = 59)			log(Carnivore)	-0.19 (0.08)	6.6	1	0.013*
				log(Initial seedbank)	0.66 (0.08)	69.7	1	<0.001***
				PesticideIntensity	-0.02 (0.05)	0.28	1	0.602
				pCrop	-0.04 (0.46)	0.01	1	0.932

Supp. Mat. Figure S5. Relationships between the log-transformed activity-density of (a) all carabid species, (b) granivore (c) omnivore and (d) seed-eating carabids at the session 1 in interaction with the log-transformed biomass of Arachnida on the log(Follow-up seedbank). Two level of the log-transformed biomass of Arachnida are represented: the mean + 1 SD (3.67 mg) and the mean – 1 SD (2.12 mg). Line is the fixed-effect prediction with the associated 95% confidence intervals, and dots are the partial residuals. A negative slope indicates a regulatory effect of the carabids on the seedbank change. The plot is created using R version 3.6.1[1] and the package 'effect'[17].

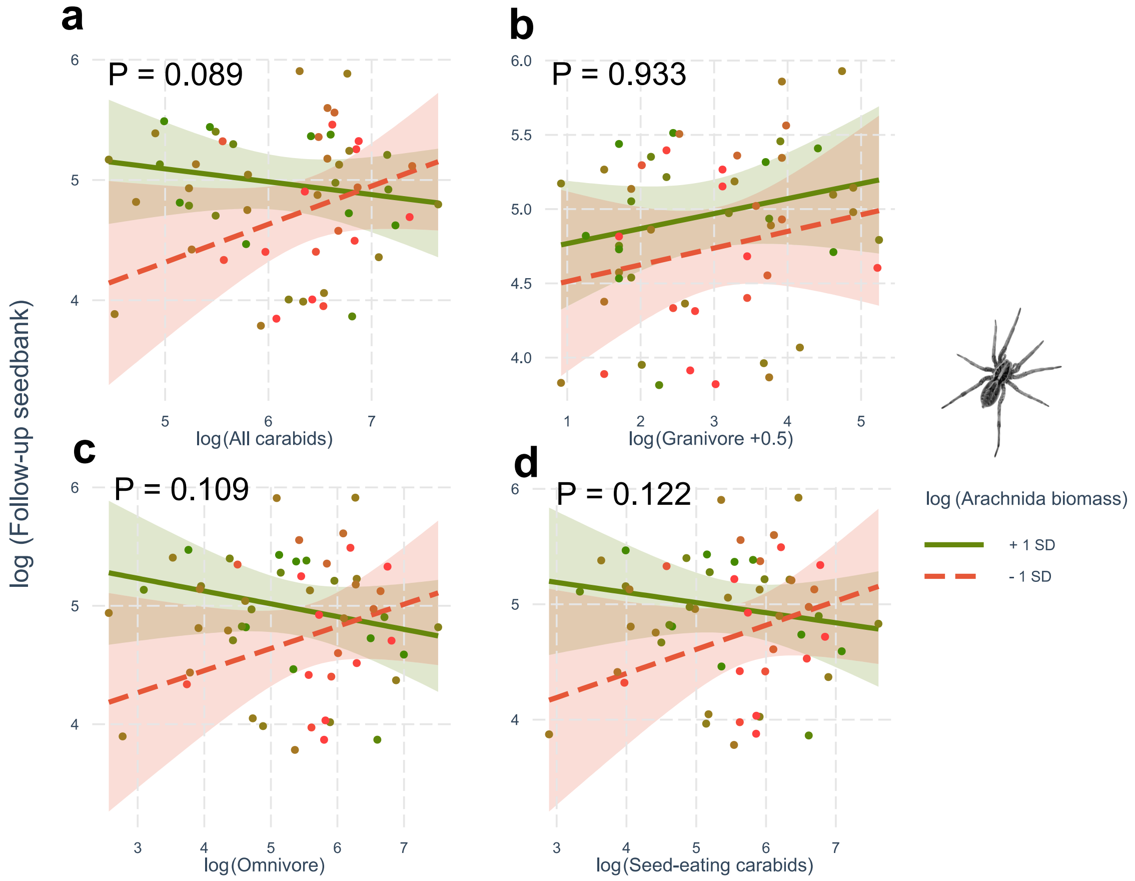

Effect of the different groups of carabids on the weed seed predation (seed card), and effect of the biomass of alternative prey on the per capita seed consumption

Supp. Mat. Table S11. Results of mixed linear models (GLMMs and LMMs) relating weed seed predation rates (based on seed cards count) to proportion of arable crops, pesticide intensity and activity-density (AD) of carabids in interaction with session. Followed by models relating the number of seed eaten per carabid to proportion of arable crops, pesticide intensity and alternative prey biomass in interaction with session. For each model we reported the type of model with the number of observations (n), AIC, marginal and condition R-squared (R^2m and R^2c), and for each variable in the model we report the estimate with standard error (Est. (Std. Error)), the wald Chi square-value (χ^2), test degrees of freedom (Df) and the P-value. Where the interaction between carabids AD or prey biomass with session was significant, we report the estimate with standard error (Est. (Std. Error)) and indicate if the slope is different from 0 (P-value) for each sampling session. For qualitative factors, such as session (S1:

session1; S2: session 2) we report the estimated marginal means (Std. Error) and an associated letter indicating the significance of the difference between the two sessions.

**Response variable (y)** **Model (n)**	**AIC**	**R²m**	**R²c**	**Explanatory variable (x)**	**Est. (Std. Error)**	**χ2**	**Df**	**P-value**
**Predation rates (seed cards)**								
GLMM (n=116)	1332.6	0.10	0.34	log(All carabids):Session	S1: 0.06 (0.14); P>0.05 ns<br>S2: 0.57 (0.16); P<0.001***	5.63	1	0.018*
				log(All carabids)	0.18 (0.19)	0.88	1	0.348
				Session	S1: 0.15 (0.02)[a]; S2: 0.08 (0.01)[b]	8.03	1	0.005**
				PesticideIntensity	-0.14 (0.07)	4.47	1	0.035*
				pCrop	0.61 (0.66)	0.85	1	0.357
GLMM (n=116)	1315.4	0.14	0.34	log(Granivores+0.5):Session	0.54 (0.09)	0.00	1	0.993
				log(Granivores+0.5)	0.54 (0.12)	18.51	1	<0.001***
				Session	S1: 0.16 (0.02); S2: 0.08 (0.01)	2.78	1	0.096.
				PesticideIntensity	0.03 (0.07)	0.23	1	0.634
				pCrop	-1.04 (0.61)	2.90	1	0.089.
GLMM (n=116)	1336.7	0.09	0.34	log(Omnivores):Session	S1: 0.06 (0.14); P>0.05 ns<br>S2: 0.57 (0.16); P=0.001***	7.01	1	0.008**
				log(Omnivores)	0.06 (0.14)	0.18	1	0.671
				Session	S1: 0.16 (0.02)[a]; S2: 0.07 (0.01)[b]	11.62	1	<0.001***
				PesticideIntensity	-0.14 (0.07)	4.05	1	0.044*
				pCrop	0.42 (0.67)	0.40	1	0.527
**Seed eaten per carabid (all species)**								
GLMM (n=116)	298.5	0.14	0.28	log(Aphid):Session	S1: -0.23 (0.10); P=0.03 *<br>S2: 0.17 (0.17); P>0.05 ns	4.80	1	0.029*
				log(Aphid)	-0.23 (0.10)	5.17	1	0.023*
				Session	S1: 0.24 (0.03)[a]; S2: 0.14 (0.02)[b]	7.17	1	0.007**
				PesticideIntensity	-0.09 (0.06)	2.11	1	0.147
				pCrop	1.04 (0.59)	3.09	1	0.079.
LMM (n=101)	293.0	0.18	0.49	log(Collembola):Session	S1: 0.09 (0.09); P>0.05 ns<br>S2: -0.19 (0.06); P=0.005**	15.80	1	<0.001***
				log(Collembola)	0.09 (0.06)	1.92	1	0.166
				Session	S1: 0.23 (0.03); S2: 0.12 (0.02)	2.01	1	0.156
				PesticideIntensity	-0.1 (0.07)	0.03	1	0.866
				pCrop	0.95 (0.70)	1.85	1	0.173
LMM (n=101)	304.5	0.09	0.21	log(Arachnida):Session	0.03 (0.78)	0.71	1	0.401
				log(Arachnida)	0.18 (0.21)	0.08	1	0.774
				Session	S1: 0.24 (0.04)[a]; S2: 0.13 (0.02)[b]	6.88	1	0.009**
				PesticideIntensity	-0.07 (0.06)	1.31	1	0.252
				pCrop	0.91 (0.63)	2.09	1	0.149
LMM (n=101)	299.4	0.14	0.27	log(Total animal):Session	-0.24 (0.11)	1.56	1	0.212
				log(Total animal)	-0.30 (0.12)	4.48	1	0.034*
				Session	S1: 0.24 (0.03)[a]; S2: 0.13 (0.02)[b]	10.07	1	0.002**
				PesticideIntensity	-0.09 (0.06)	2.20	1	0.138
				pCrop	1.01 (0.59)		1	0.089.
**Seed eaten per granivore**								
LMM (n=101)	303.4	0.19	0.55	log(Aphid):Session	-0.14 (0.09)	0.02	1	0.894
				log(Aphid)	-0.14 (0.10)	2.33	1	0.127
				Session	S1: 5.74 (0.8)[a]; S2: 3.10 (0.5)[b]	13.63	1	<0.001***
				PesticideIntensity	0.17 (0.07)	5.21	1	0.022*
				pCrop	-1.63 (0.70)	5.47	1	0.019*
LMM (n=101)	304.7	0.18	0.50	log(Collembola):Session	0.05 (0.06)	0.32	1	0.572
				log(Collembola)	0.07 (0.07)	0.62	1	0.431
				Session	S1: 5.59 (0.8)[a]; S2: 3.18 (0.5)[b]	10.3	1	0.001**
				PesticideIntensity	0.19 (0.07)	6.36	1	0.012*
				pCrop	-1.42 (0.74)	3.66	1	0.056.
LMM (n=101)	305.5	0.17	0.52	log(Arachnida):Session	-0.01 (0.11)	0.13	1	0.723
				log(Arachnida)	-0.08 (0.24)	0.00	1	0.952
				Session	S1: 5.66 (0.86)[a]; S2: 3.15 (0.5)[b]	8.88	1	0.003**
				PesticideIntensity	0.20 (0.07)	8.50	1	0.004**
				pCrop	-1.75 (0.71)	6.02	1	0.014*
LMM (n=101)	302.2	0.19	0.56	log(Total animal):Session	-0.22 (0.11)	0.00	1	0.987
				log(Total animal)	-0.22 (0.12)	3.68	1	0.055.
				Session	S1: 5.79 (0.84)[a]; S2: 3.08 (0.50)[b]	14.50	1	<0.001***

				PesticideIntensity	0.18 (0.07)	6.77	1	0.009**
				pCrop	-1.77 (0.69)	6.54	1	0.011*
**Seed eaten per omnivore**								
LMM (n=101)	326.2	0.20	0.45	log(Aphid):Session	S1: -0.34 (0.11); P=0.003** S2: 0.07 (0.19); P>0.05 ns	4.32	1	0.038*
				log(Aphid)	-0.34 (0.11)	9.22	1	0.002**
				Session	S1: 0.60 (0.09)[a]; S2: 0.24 (0.04)[b]	8.07	1	0.005**
				PesticideIntensity	-0.04 (0.08)	0.31	1	0.578
				pCrop	0.99 (0.73)	1.85	1	0.174
LMM (n=101)	313.1	0.26	0.69	log(Collembola):Session	S1: 0.18 (0.08); P=0.015* S2: -0.21 (0.07); P=0.005 **	35.12	1	<0.001***
				log(Collembola)	0.18 (0.07)	6.12	1	0.013*
				Session	S1: 0.55 (0.09); S2: 0.19 (0.03)	3.18	1	0.075.
				PesticideIntensity	0.06 (0.08)	0.50	1	0.48
				pCrop	0.86 (1.32)	1.74	1	0.187
LMM (n=101)	335.0	0.14	0.35	log(Arachnida) :Session	-0.04 (0.13)	0.62	1	0.433
				log(Arachnida)	0.12 (0.24)	0.09	1	0.767
				Session	S1: 0.56 (0.10)[a]; S2: 0.23 (0.05)[b]	13.22	1	<0.001***
				PesticideIntensity	0.02 (0.08)	0.06	1	0.801
				pCrop	-0.23 (0.29)	1.00	1	0.317
LMM (n=101)	329.0	0.18	0.40	log(Total animal):Session	-0.33 (0.13)	0.66	1	0.416
				log(Total animal)	-0.38 (0.14)	6.37	1	0.012*
				Session	S1: 0.59 (0.09)[a]; S2: 0.22 (0.04)[b]	22.44	1	<0.001***
				PesticideIntensity	-0.02 (0.07)	0.06	1	0.801
				pCrop	0.86 (0.72)	1.43	1	0.231

Maps and sampling design:

Supp. Mat. Figure S6. Location of the sampling sites at the European scale for each country (Austria, the Czech Republic, France and Sweden). The map was regenerated using R version 3.6.1[1] and the package "rnaturalearth"[18] version 0.1.0 (https://CRAN.R-project.org/package=rnaturalearth).

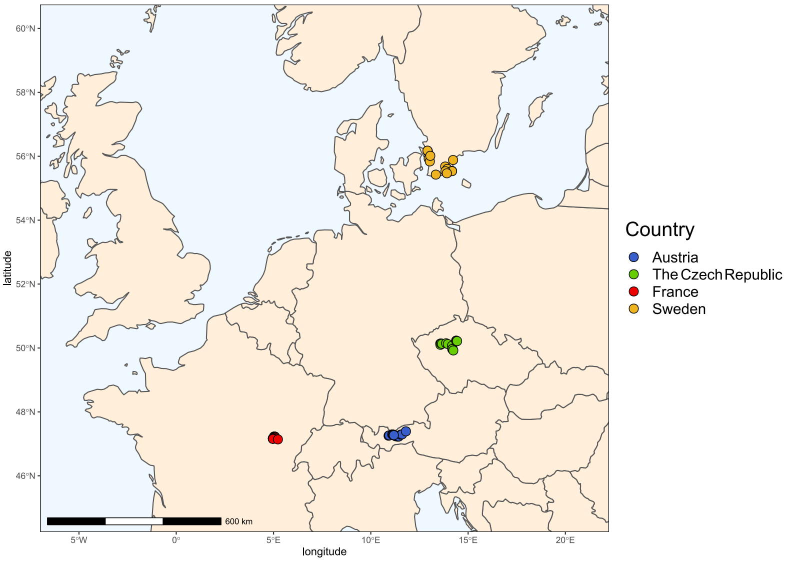

Supp. Mat. Table S12. Coordinates of the sampling sites for each country

Country	Latitude	Longitude
Austria	47°15'27.65"N to 47°23'44.74"N	10°54'57.06"E to 11°48'27.11"E
The Czech Republic	49°55'30.07"N - 50°13'59.66"N	13°34'12.18"E - 4°26'21.91"E
France	47° 8'33.72"N - 47°14'2.40"N	4°57'59.04"E - 5°13'39.00"E
Sweden	55°25'44.33"N - 56°10'44.33"N	12°54'59.44"E - 14°10'34.02"E

Supp. Mat. Figure S7. Schematic representation of the sampling plan of a field. The left side represents the theoretical locations of the four transects within the field. The right side represents the arrangement of measurements (Vortis sampling, pitfall traps, soil samples for seedbank evaluation and seed cards) along a transect.

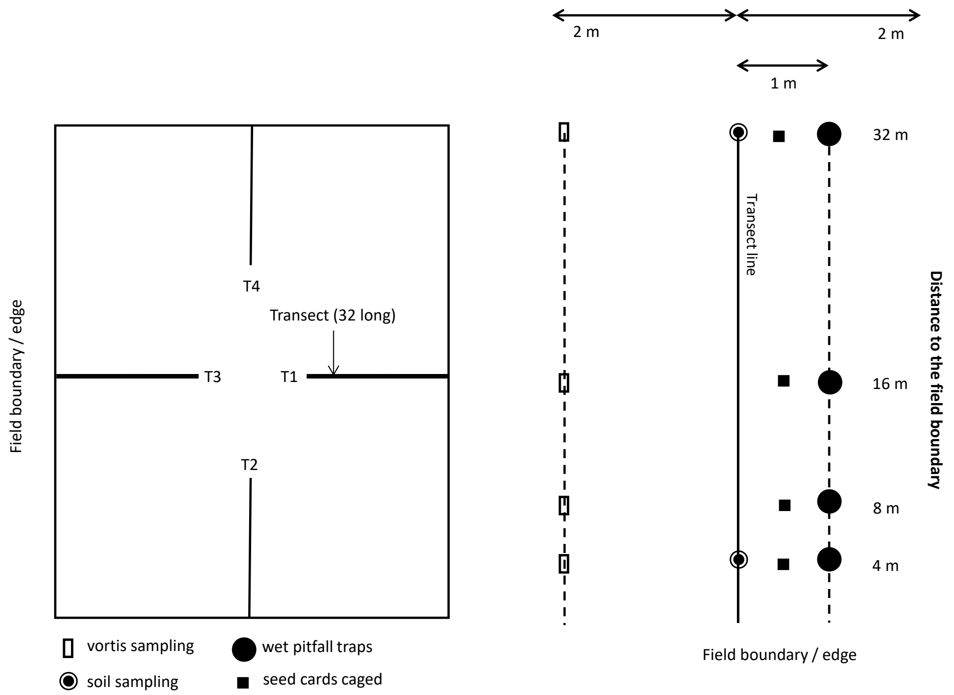

Supp. Mat. Figure S8. Proportion of arable crops in the landscape against pesticide intensity, with associated boxplot for the 57 fields with colours indicating the country. The plot is created using R version 3.6.1[1] and the package 'ggpubr'[11].

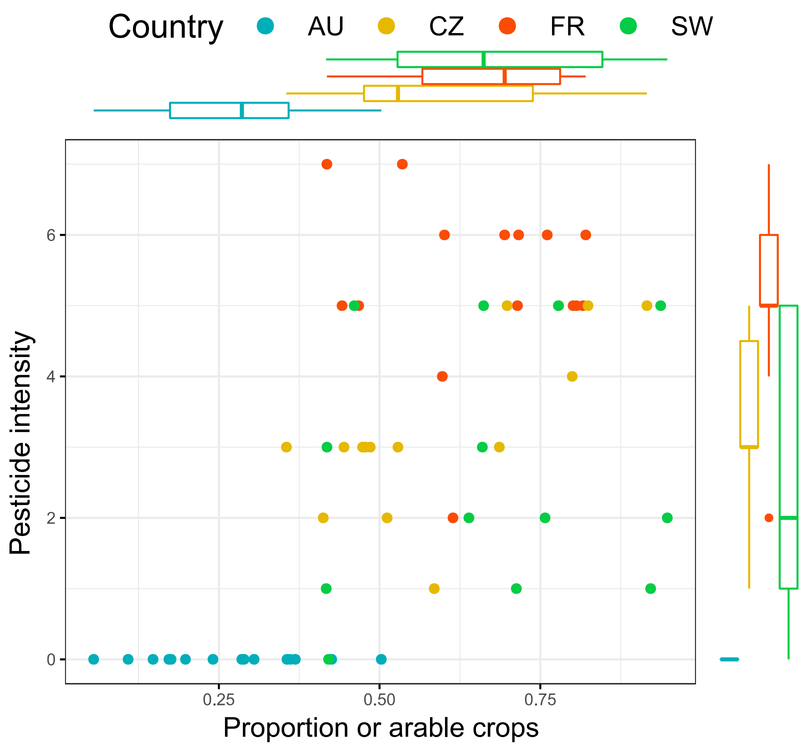

Supp. Mat. Table S13. Descriptive statistics (mean (SD), median [min, max] and sum) for field size, number of field visit to spray pesticide (Pesticide Intensity) and the proportion of arable crops (pCrop)

	**AU (n=15)**	**CZ (n=15)**	**FR (n=15)**	**SW (n=15)**	**Overall (n=60)**
**PesticideIntensity**					
Mean (SD)	0.00 (0.00)	3.33 (1.23)	5.33 (1.23)	2.69 (1.80)	2.84 (2.29)
Median [Min, Max]	0.00 [0.00, 0.00]	3.00 [1.00, 5.00]	5.00 [2.00, 7.00]	2.00 [0.00, 5.00]	3.00 [0.00, 7.00]
Missing	0 (0%)	0 (0%)	0 (0%)	2 (13.3%)	2 (3.3%)
**pCrop**					
Mean (SD)	0.266 (0.124)	0.598 (0.172)	0.654 (0.141)	0.683 (0.195)	0.550 (0.230)
Median [Min, Max]	0.286 [0.0554, 0.503]	0.529 [0.355, 0.916]	0.695 [0.418, 0.821]	0.662 [0.417, 0.947]	0.532 [0.0554, 0.947]

Supp. Mat. Figure S9. Schematic representation of the expectation tested. We organized the tests of our hypotheses as four expectations: (i) there is a negative relationship between the weed seedbank change and the activity-density (AD) of carabids; (ii) the observed weed seedbank regulation relationship co-varies with the alternative prey biomass; (iii) the relationship between the AD of carabids and weed seed predation will be significant and positive; and (iv) the alternative prey biomass will be a significant co-variate of the per capita seed consumption by carabids. Red arrows indicate a negative expected effect, green arrows a positive effect, and yellow arrows an interaction effect.

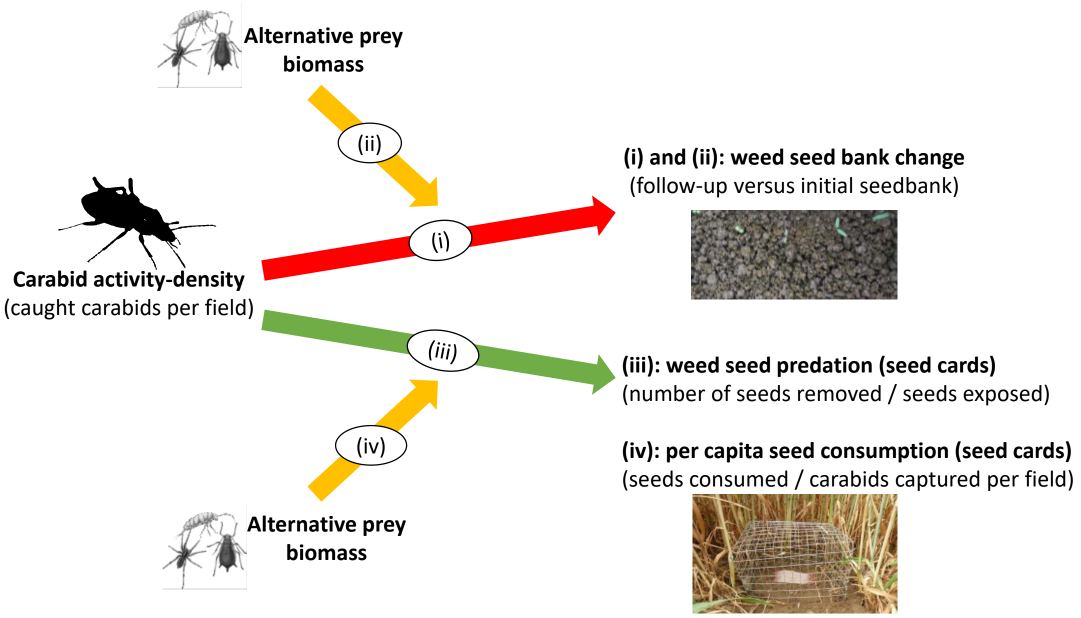

Correlation between the different groups of carabids and the biomass of the different alternative prey groups

Supp. Mat. Table S14. Verification of autocorrelation between carabids and prey variables using LMM with country and session in fixed effect, and field in random effect. We report the estimates and the p-value.

	log(All carabids)	log(Granivore + 0.5)	log(Omnivore)	log(Seed-eating)	log(Aphididae biomass)	log(Arachnida biomass +0.5)	log(Collembola biomass + 0.5)	log(All prey biomass)	Predation rate
log(All carabids)		0,29 (0)	0,71 (0)	0,86 (0)	0,05 (0,4)	-0,08 (0,3)	-0,06 (0,111)	-0,03 (0,681)	0,91 (0,022)
log(Granivore + 0.5)	0,57 (0)		0,26 (0,01)	0,49 (0)	-0,04 (0,669)	-0,11 (0,223)	-0,13 (0,034)	-0,13 (0,186)	2,46 (0)
log(Omnivore)	1,13 (0)	0,21 (0,009)		1,11 (0)	0,14 (0,094)	-0,03 (0,762)	-0,11 (0,027)	0,03 (0,746)	0,62 (0,218)
log(Seed-eating)	1,01 (0)	0,3 (0)	0,83 (0)		0,13 (0,083)	-0,03 (0,733)	-0,11 (0,011)	0,03 (0,776)	0,88 (0,042)
log(Aphididae biomass)	0,11 (0,472)	-0,01 (0,953)	0,18 (0,132)	0,21 (0,117)		-0,01 (0,91)	-0,1 (0,086)	1,11 (0)	-1,42 (0,023)
log(Arachnida biomass +0.5)	-0,13 (0,309)	-0,09 (0,32)	-0,03 (0,777)	-0,04 (0,71)	-0,05 (0,561)		0,08 (0,106)	0,03 (0,771)	0,01 (0,989)
log(Collembola biomass + 0.5)	-0,06 (0,709)	-0,19 (0,203)	-0,12 (0,344)	-0,12 (0,391)	0,14 (0,208)	0,36 (0)		0,4 (0)	-1,94 (0)
log(All prey biomass)	-0,05 (0,702)	-0,07 (0,441)	0,05 (0,601)	0,06 (0,593)	0,73 (0)	0,04 (0,673)	0,01 (0,892)		-1,82 (0)
Predation rate	0,05 (0,027)	0,07 (0)	0,02 (0,323)	0,04 (0,048)	-0,02 (0,1)	0 (0,931)	-0,01 (0,3)	-0,06 (0,001)	

Supp. Mat. Figure S10. Correlation of the carabids (seed-eating, omnivore, granivore) and preys variables (Aphididae, Collembola and Arachnida) with the first two dimensions of a Principal Component Analysis (PCA). With the representation of the 120 field-session classified per countries according to their colour and shape. The plot is created using R version 3.6.1[1] and the package 'factoextra'[19].

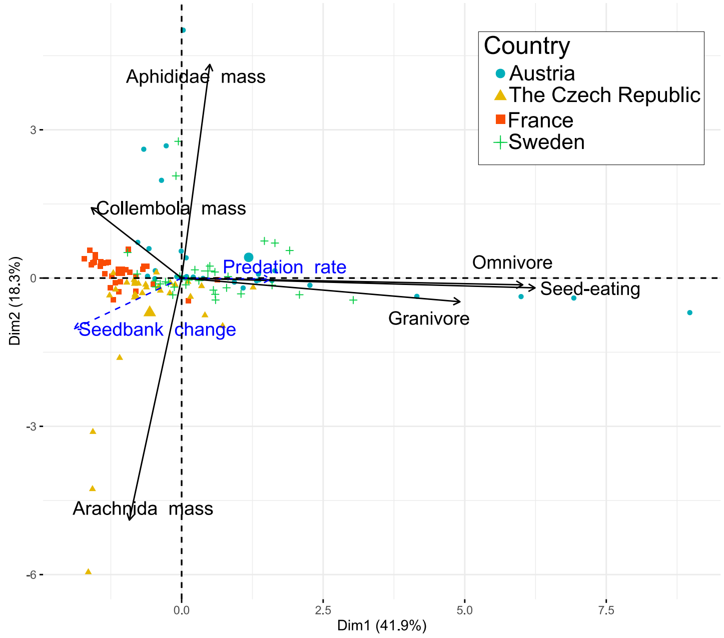

References

1. R Core Team. R: A language and environment for statistical computing. *R Foundation for Statistical Computing* https://www.R-project.org (2019).

2. Wickham, H. *ggplot2: Elegant Graphics for Data Analysis*. (Springer-Verlag New York, 2016).

3. Homburg, K., Homburg, N., Schäfer, F., Schuldt, A. & Assmann, T. Carabids.org - a dynamic online database of ground beetle species traits (Coleoptera, Carabidae). *Insect Conserv. Divers.* **7**, 195–205 (2014).

4. Fournier, B., Gillet, F., Le Bayon, R. C., Mitchell, E. A. D. & Moretti, M. Functional responses of multitaxa communities to disturbance and stress gradients in a restored floodplain. *J. Appl. Ecol.* **52**, 1364–1373 (2015).

5. Purtauf, T., Dauber, J. & Wolters, V. The response of carabids to landscape simplification differs between trophic groups. *Oecologia* **142**, 458–464 (2005).

6. Saska, P., Honěk, A. & Martinková, Z. Preferences of carabid beetles (Coleoptera: Carabidae) for herbaceous seeds. *Acta Zool. Acad. Sci. Hungaricae* **65**, 57–76 (2019).

7. Saska, P., Van Der Werf, W., De Vries, E. & Westerman, P. R. Spatial and temporal patterns of carabid activity-density in cereals do not explain levels of predation on weed seeds. *Bull. Entomol. Res.* **98**, 169–181 (2008).

8. Honěk, A., Martinkova, Z., Saska, P. & Pekar, S. Size and taxonomic constraints determine the seed preferences of Carabidae (Coleoptera). *Basic Appl. Ecol.* **8**, 343–353 (2007).

9. Honěk, A., Martinkova, Z. & Jarosik, V. Ground beetles (Carabidae) as seed predators. *Eur. J. Entomol.* **100**, 531–544 (2003).

10. Koprdova, S., Saska, P. & Soukup, J. The spectrum of invertebrate seed predators that contribute to the control of the rape volunteer seeds (Brassica napus L.). *J. Plant Dis. Prot.* 261–264 (2008).

11. Kassambara, A. ggpubr: 'ggplot2' Based Publication Ready Plots. R package version 0.2.4 (2019).

12. Pey, B. *et al.* A Thesaurus for Soil Invertebrate Trait-Based Approaches. *PLoS One* **9**, e108985 (2014).

13. Nentwig, W., Blick, T., Gloor, D., Hänggi, A. & Kropf, C. Araneae: Spiders of Europe. *https://araneae.nmbe.ch*.

14. Penell, A., Raub, F. & Höfer, H. Estimating biomass from body size of European spiders based on regression models. *J. Arachnol.* **46**, 413 (2018).

15. Caballero, M., Baquero, E., Ariño, A. H. & Jordana, R. Indirect biomass estimations in Collembola. *Pedobiologia (Jena).* **48**, 551–557 (2004).

16. Migui, S. M. & Lamb, R. J. Sources of variation in the interaction between three cereal aphids (Hemiptera: Aphididae) and wheat (Poaceae). *Bull. Entomol. Res.* **96**, 235–241 (2006).

17. John, F. & Weisberg, S. *An R Companion to Applied Regression*. (Sage, 2019).

18. South, A. rnaturalearth: World Map Data from Natural Earth. R package version 0.1.0 (2017).

19. Kassambara, A. & Mundt, F. factoextra: Extract and Visualize the Results of Multivariate Data Analyses. R package version 1.0.5 (2017).
